# Targeting Lipid Metabolism in Cancer Stem Cells for Anticancer Treatment

**DOI:** 10.3390/ijms252011185

**Published:** 2024-10-17

**Authors:** Manish Kumar Singh, Sunhee Han, Sungsoo Kim, Insug Kang

**Affiliations:** 1 Department of Biochemistry and Molecular Biology, School of Medicine, Kyung Hee University, Seoul 02447, Republic of Korea; manishbiochem@gmail.com (M.K.S.); sunheehan@khu.ac.kr (S.H.); 2 Biomedical Science Institute, Kyung Hee University, Seoul 02447, Republic of Korea; 3 Department of Biomedical Science, Graduate School, Kyung Hee University, Seoul 02447, Republic of Korea

**Keywords:** autophagy, cancer stem cell, drug resistance, lipids, stemness, tumor microenvironment

## Abstract

Cancer stem cells (CSCs), or tumor-initiating cells (TICs), are small subpopulations (0.0001–0.1%) of cancer cells that are crucial for cancer relapse and therapy resistance. The elimination of each CSC is essential for achieving long-term remission. Metabolic reprogramming, particularly lipids, has a significant impact on drug efficacy by influencing drug diffusion, altering membrane permeability, modifying mitochondrial function, and adjusting the lipid composition within CSCs. These changes contribute to the development of chemoresistance in various cancers. The intricate relationship between lipid metabolism and drug resistance in CSCs is an emerging area of research, as different lipid species play essential roles in multiple stages of autophagy. However, the link between autophagy and lipid metabolism in the context of CSC regulation remains unclear. Understanding the interplay between autophagy and lipid reprogramming in CSCs could lead to the development of new approaches for enhancing therapies and reducing tumorigenicity in these cells. In this review, we explore the latest findings on lipid metabolism in CSCs, including the role of key regulatory enzymes, inhibitors, and the contribution of autophagy in maintaining lipid homeostasis. These recent findings may provide critical insights for identifying novel pharmacological targets for effective anticancer treatment.

## 1. Introduction

Cancer is a multifactorial disease and ranked as the second cause of death in the United States in 2020 and 2021 [1]. The National Cancer Institute GDC Data Portal reports an increasing number of cases every year, with the highest cases being bone marrow and blood cancer, followed by lung cancer. While advanced-stage cancer offers bleak prospects for complete recovery and reduces survival rates, early detection, accurate diagnosis, and effective treatment are crucial for improving a patient’s quality of life and increasing survival rates. Despites advancements in treatment, recurrence and relapse rates remain high, largely due to tumor heterogeneity and therapy resistance. CSCs have been identified as significant contributors to drug resistance and tumor recurrence.

CSCs use alternative sources of energy and altered metabolism to thrive in the tumor microenvironment [2]. They utilize substrates, such as glutamine, serine, and fatty acids in addition to glucose, to accomplish the energy requirement [3]. Alterations in lipid metabolism have been linked to cancer development, particularly in obese individuals. Studies have shown de novo lipid synthesis facilitates resistance to oxidative stress, and targeting them makes cancer cells susceptible to chemotherapy [4].

Autophagy is a key cellular pathway for protein degradation and nutrient recycling to maintain cellular homeostasis. Induced autophagy has been reported in CSCs, enabling them to adapt to the tumor microenvironment. Targeting these pathways, including lipid metabolism, opens avenues for identifying new targets for cancer treatment and shedding light on potential therapeutic interventions.

## 2. Cancer Stem Cells (CSCs)

**Figure 1:**
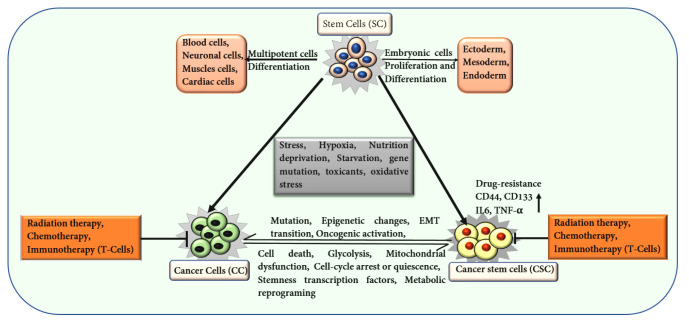
Illustration depicting the characteristics of stem cells (SCs), cancer cells (CCs), and CSCs. The transformation of SCs into CCs and CSCs is influenced by various factors and conditions, which subsequently support cancer cell self-renewal and tumor recurrence. This image highlights associations of CCs and CSCs with various processes, such as stress, chemo and radiation therapy, and metabolic reprogramming, which contribute to CC progression, CSC self-renewal, and metastasis.

CSCs represent a distinct and specialized subset of cells, typically comprising less than 1% of the total cancer cell population within tumors. These cells exhibit stem cell properties, including an enhanced capacity for self-renewal and the ability to differentiate into various tumorigenic cell types. CSCs are notably resistant to conventional therapies and play a pivotal role in tumor initiation, progression, and metastasis [5]. In 1997, John and Bonnet first identified cells with significant proliferative properties in acute myeloid leukemia (AML). They successfully isolated CSCs marked by the surface marker CD34^+^ and CD38^−^ [6]. The identification of these leukemia stem cells support the theory that CSCs, with self-renewing potential, derive tumorigenesis, akin to normal stem cells but with a pathological role in tumor progression [7]. Unlike normal stem cells, which possess a spectrum of differentiation potentials ranging from totipotency to unipotency [8], CSCs are integral to the malignant process, promoting the growth and spread of cancer. An enhanced expression of cell surface markers, such as CD44, CD36, CD90, CD133, and partner of SLD five 1 (PSF1) [9], transition into a dormant or quiescent state support stemness and drug resistance in CSCs (Figure 1) [10]. Hypoxia conditions and activation of specific genes related to epithelial-to-mesenchymal cell transition (EMT) strengthen the metastasis [11]. EMT is crucial for cancer cell metastasis and resistance to anoikis, a type of programmed cell death triggered by the detachment of epithelial cells. CSCs recruit immune cells, such as tumor-associated neutrophils (TAN), tumor-associated macrophages (TAM), and myeloid-derived suppressor cells (MDSC), that facilitate immunosuppression and support metastasis [12]. Various immune molecules, including IL-6, IL-4, IL-8, TGF-β, and TNF-α, support CSC proliferation and growth, aiding in CSC stemness in various cancers, such as breast, liver, oral squamous, and lung cancer (Figure 1) [13,14].

**Figure 2:**
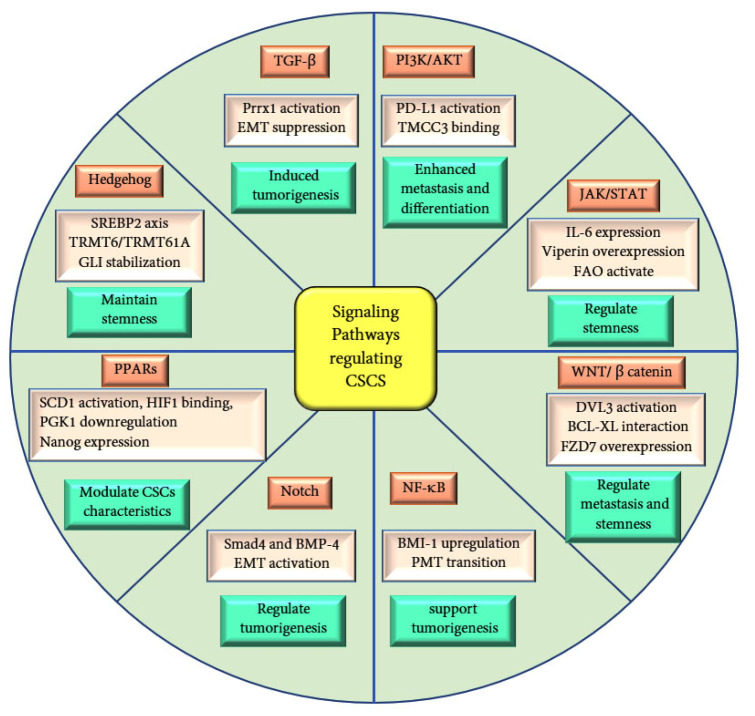
Schematic representation of the key signaling pathways involved in regulating CSCs. The figure highlights critical factors within each pathway that govern CSC characteristics, including stemness, proliferation, survival, and drug resistance. These pathways play a pivotal role in maintaining CSC functions and contribute to cancer progression and therapeutic resistance.

CSCs are regulated by various signaling pathways that are crucial for maintaining stemness and contribute to tumor progression. Peroxisome proliferator-activated receptors (PPARs), nuclear receptors involved in fat and glucose metabolism, play a critical role in modulating CSC characteristics. PPARs have three subtypes: PPARα, PPARδ, and PPARγ. In liver CSCs, activation of the PPARα pathway and enrichment of its downstream effector, stearoyl-CoA desaturase 1 (SCD1), is crucial for maintaining stemness [15]. The PPAR pathway also influences CSC traits through glucose metabolism. For instance, low PPARα expression in AML CSCs is inversely correlated with stemness. PPARα inhibits glucose metabolism by binding to HIF1α, reducing the expression of its downstream phosphoglycerate kinase 1 (PGK1) gene, thereby suppressing CSC stemness [16]. In hepatic CSCs, fatty acid 4-phenylbutyric acid (4-PBA) upregulates PPARα expression, preventing its degradation and promoting CSC initiation and tumorigenicity [17]. N1-methyladenosine methylation enhances PPARδ expression in hepatic CSCs, activating the PPAR pathway to regulate cholesterol metabolism, sustain stemness, and increase tumorigenicity [18]. PPARδ activation is also linked to high-fat, diet-induced colorectal cancer liver metastasis via upregulation of Nanog (Figure 2) [19]. Chen et al. reported that Nanog, a key regulator of stem cell fate, promotes the generation of stem-like tumor-initiating cells (TICs) and drives hepatocellular carcinoma (HCC) oncogenesis through metabolic reprogramming, specifically by shifting from oxidative phosphorylation (OXPHOS) to FAO [20]. Conversely, PPARγ activation inhibits migration in glioma stem cells (GSCs), reduces stemness in brain CSCs by downregulating SOX2 and YAP1, and promotes differentiation by inducing COL2A1 and HLXB9 expression [21,22].

Wnt/β-Catenin signaling is implicated in various cancers, including lung, liver, thyroid, colorectal, cervical, and glioblastoma. In the canonical Wnt signaling pathway, Wnt ligands bind to frizzled transmembrane receptors, activating disheveled, which interact with the T-cell factor/LEF family to stabilize and accumulate nuclear β-catenin, promoting transcriptional activity. During tumorigenesis, Wnt signaling enhances tumor migration and invasion by upregulating genes involved in cell adhesion, such as Eph/ephrin, E-cadherin, and matrix metalloproteinases (MMPs), thereby supporting stemness in CSCs [23]. In colorectal cancer cells, this pathway strongly derives tumor-initiating effects in CD44+ and CD133+ CSCs [24]. Farnesyl Dimethyl Chromanol (FDMC), a Wnt/β-Catenin pathway inhibitor, reduces stemness and metastatic potential in colorectal CSCs via inducing apoptosis [25]. Upregulation of Disheveled3 (DVL3) activates the Wnt/β-Catenin/c-Myc/SOX2 axis, enhancing CSC stemness and metastatic in colorectal cancer [26]. In gastric cancer, the ST2^+^ marker promotes metastasis via Wnt pathway activation and interaction with BCL-XL [27]. Similarly, in pancreatic cancer, elevated expression of Frizzled-7 (FZD7) derives the CSC phenotype and liver metastasis through the canonical Wnt/β-Catenin pathway (Figure 2) [28].

Hedgehog (Hh) has been implicated in the tumor-initiating function of CSCs. In hepatic CSCs, the Hh pathway is maintained by the caspase-3/Sterol-regulatory element-binding Protein-2 (SREBP2) axis, which supports cholesterol synthesis, stemness, and tumorigenicity. TRNA methyltransferase 6 (TRMT6)/TRMT61A-mediated N1-methyadenosine methylation in liver CSCs promotes cholesterol metabolism and activates the Hh pathway. Maintaining stemness also enhances tumorigenicity (Figure 2). Hh signaling is further involved in maintaining the breast CSCs through the stabilization of GLI1 transcription [29].

The Notch pathway plays a significant role in CSC metastasis in various cancers, including breast, glioma, renal, and ovarian cancers. In breast cancer, Bone Morphogenetic Protein 4 (BMP-4) enhances stemness and EMT programs by activating the Notch pathway in a Smad4-dependent manner [30]. Upregulated Notch signaling in renal CSCs contributes to malignant behaviors, such as metastasis, stemness maintenance, and tumorigenesis [31]. Continuous activation of the Notch pathway leads to overexpression of CSC marker genes, resulting in enhanced tumorigenesis compared to cells with normal Notch activity [32].

The NF-κB pathway is crucial for maintaining CSC stemness and aggressive metastasis. In glioma stem cells (GSCs), NF-κB activation supports the proneuronal to mesenchymal transition (PMT) [33]. In CD133^+^ liver CSCs, BMI-1-induced NF-κB activation and nuclear translocation promote CSC stemness and metastatic potential while inhibiting apoptosis [34]. Ovarian CSCs exhibit high expression of NF-κB pathway-related proteins, and inhibiting the NF-κB reduces the CSC population [35]. In breast cancer, elevated expression of stromal cell-derived factor-1 (SDF-1) induces stemness and EMT by activating the NF-κB pathway (Figure 2) [36]. In colon cancer inhibition of NF-κB, using dimethylamine parthenolide (DMAPT) altered the metabolomic profiles, reduced unsaturated lipid levels, and impaired spheroid formation in colon CSCs, underscoring this pathway in preserving CSC stemness [37,38].

The JAK/STAT pathway is involved in maintaining CSC stemness across various cancers. In breast cancer, inhibiting the JAK/STATE reduces the expression of stemness-related genes [39]. Activation of the lipid metabolism-related STAT3/CPT1B/fatty acid β-oxidation (FAO) axis in breast CSCs is positively correlated with stemness maintenance [40]. Immunity plays a crucial role in activating the JAK/STAT pathway in CSCs, as Il-6 secreted by regulatory T-cells induces the STAT3 pathway in glioma cells, sustaining the stemness phenotype (Figure 2) [41]. In prostate CSCs, IL-6 promotes tumorigenesis, and its inhibition eliminates tumor initiation. The interferon-inducible (IFN-inducible) protein viperin overexpression in CSCs partially inhibits FAO through the JAK/STAT pathway, reprogramming metabolism to activate tumor progression. However, viperin-expressing cells have more accumulation of LDs, compared to cells with basal level of expression [42].

The TGF-β pathway is closely linked to CSC tumorigenesis and stemness. In various cancers, including ovarian and oral squamous carcinoma, inhibiting the TGF-β pathway suppresses CSC stemness and EMT [43,44]. Paired-Related Homeobox 1 (Prrx1) activates the TGF-β/Smad pathway in GSCs, inducing stemness and promoting vascularization and therapeutic resistance (Figure 2) [45].

The PI3K/AKT pathway is pivotal in driving the differentiation of normal stem cells into CSCs in various cancers [46]. In breast cancer, continuous activation of PI3K/AKT pathway by PD-L1 is crucial for maintaining CSC stemness (Figure 2) [47]. In breast CSCs, the transmembrane and coiled-coil domain family 3 (TMCC3) activates PI3K/AKT via binding to AKT, promoting tumorigenesis and metastasis (Figure 2) [48]. In liver cancer, the tumor suppressor Connexin 32 (Cx32) attenuates PI3K/AKT pathway activity, suppressing stemness and tumorigenicity [49]. Stress-induced phosphoprotein 1 (STIP1) enhances MMP2 and MMP-9 expression in osteosarcoma by activating the PI3K/AKT and ERK1/2 pathways, ultimately promoting CSC metastasis [50].

CSCs undergo metabolic reprogramming to sustain proliferation and survival, particularly under nutritional deficiency. They primarily rely on aerobic glycolysis (Warburg effect) [51] and adjust their metabolism to meet their energy requirements [52]. Reprogrammed lipid metabolism, including lipid uptake, de novo lipogenesis, fatty acid activation, desaturation, chain elongation, FAO, and lipid droplet (LD) storage and degradation, supports cancer growth and proliferation [53], enabling CSCs to evade cell death and promotes cancer progression [54]. Mitochondrial metabolism is vital for fulfilling the energy demands necessary for sustained CSC proliferation while also supplying substrate required by various cellular compartments. Studies have shown that poorly differentiated counterparts demonstrate increased glycolytic activity, primarily due to impaired differentiation in CSCs [55]. In breast CSCs, the overexpression of pyruvate dehydrogenase kinase 1 (PDK1) suppresses mitochondrial glycolysis. The depletion of PDK1 levels significantly decreased the population of ALDH1-positive BCSCs, hindering their ability to form spheroids [55].

Additionally, CSCs display high levels of unsaturated fatty acids, and inhibiting key enzymes, such as stearoyl-COA desaturase 1 (SCD1) and acetaldehyde dehydrogenase 1 A1 (ALDH1A1), prevents apoptosis and enhances stemness [56]. Elevated ALDH1 levels are linked to various cancers, including lung cancer [57], breast cancer [58], esophageal cancer [59], and colon cancer [60], where it contributes to enhance their stemness and tumorigenicity. ALDH1A1 inhibitor (CM037) significantly decreased levels of unsaturated lipids and suppressed spheroid forming of CSCs [38]. SCD1 inhibitor, CAY10566, effectively reduced unsaturated lipid levels in CSCs and inhibited spheroid formation, suggesting that SCD1 activity is essential for CSC stemness and tumorigenicity [38]. These findings suggest that glycolytic intermediates may be redirected to fuel increased lipogenesis in CSCs, further enhancing their stemness. Therefore, targeting intermediates within the glycolytic pathway may provide a promising strategy to eliminate CSCs, reducing tumor growth, metastasis, and recurrence in cancer patients [61].

In ovarian cancer, CSCs prefer fatty acid β-oxidation over the Warburg effect [62]. Glioblastoma CSCs (GBM-CSCs) express high levels of fatty acid synthase (FASN), and inhibiting FASN reduces their proliferation and migration [63]. In head and neck squamous cell carcinoma, ALDH^+^ cells inhibit T-cell proliferation and secrete cytokines, such as interferon γ (IFN-γ), IL-2, and tumor necrosis factor-α (TNF-α), promoting tumorigenesis and drug resistance [64]. Orlistat has been shown to inhibit FASN activity in CRC cell lines (CaCo-2 and SW480) [65]. In vivo studies with HT-29/tk-luc human colorectal carcinoma-bearing animal models have demonstrated Orlistat’s capacity to suppress tumor growth [66]. Additionally, novel FASN inhibitors, TVB-3166 and TVB-3664, currently in Phase II clinical trials, significantly reduced tumor volume in patient-derived CRC xenograft models [67]. Natural compounds like (E)-N-(2-(4-methoxystyryl) phenyl) furan-2-carboxamide have also shown promising chemo-preventive activity in HCT116 cells [68]. Further, investigation of the combined effects of ALDH and FASN inhibitors, as well as their derivatives, across various cancers is crucial to explore their potential in preventing cancer progression by inhibiting CSC growth and progression.

## 3. Lipid Metabolism in CSCs

Lipid metabolism has been associated with several metabolic diseases and pathological cancers. Recent evidence has shown lipid metabolism is crucial for maintenance of CSCs properties. CSCs exhibit elevated LDs compared to normal cells. During metabolic stress resulting from inhibiting glycolysis, free fatty acids (FFAs) from LDs are used for ATP production through FAO. Dysregulation of lipids poses a significant risk for cancer development, particularly in a condition known as cachexia, characterized by severe weight loss, physical deterioration, loss of muscles and adipose tissue, anorexia, inflammation, and insulin resistance [69]. Lipolysis in adipose tissue is associated with tumor progression by supplying energy to cancer cells [70]. For instance, increased levels of circulating free fatty acids, mono-acylglycerides, and di-acylglycerides have been observed in cachectic ovarian cancer patients [71].

Increased uptake of lipids contributes to the accumulation of LDs, thereby enhancing the tumor-initiating capacity of CSCs [72]. Elevated lipid content in colorectal cancer stem cells (CRCSCs) has been associated with greater clonogenicity and tumorigenicity [73]. Notably, the inhibition of phospholipase A2 leads to a reduction in LDs and triggers apoptosis [74].

Regulation of the transport of long-chain fatty acids into the mitochondria for FAO is mediated by changes in the expression of carnitine palmitoyl transferase 1A (CPT1A) (Table 1). In colorectal CSCs, increased LDs serve as unique markers and are induced by hypoxia via HIF-1 and HIF-2 mediated repression of CPT1A [75]. Increased CPT1A expression promotes tumor progression and metastasis by inhibiting cell death (apoptosis) or detachment-induced cell death (anoikis) in breast cancer and leukemia [76]. CPT1A is involved in a positive feedback loop with cMyc. For instance, CPT1A-cMyc loop predominantly suppresses tumoral ferroptosis via NRF2/GPX4 and CSL4/PUFA-PLs pathways in lung cancer [77]. However, the molecular interaction of c-Myc and CPT1A in FAO and PUFA-PLs loop is poorly understood. Thus, the amount of various lipid species and fatty acids, which are basic constituents of lipid membranes and key players in signal transduction pathways, has revealed a significant role in the maintenance of CSC stemness [78].

CSCs are characterized by a high content of unsaturated lipids, including monounsaturated fatty acids (MUFAs), which have been shown to influence CSC characteristics [79]. The enzyme SCD1 facilitates desaturation and is critical for CSC generation and maintenance in various cancers, such as ovarian, breast, and liver cancers [37,80]. In colorectal CSCs, SCD1 and ALDH1A1 significantly contribute to maintaining stemness [38]. Inhibition of desaturases in ovarian cancer reduces cancer stemness, thereby increasing their susceptibility to treatment [37]. Sterol regulatory element binding proteins (SREBPs), a family of helix-loop-helix leucine zipper transcription factors, play a pivotal role in the regulation of lipogenesis. They induce the biosynthesis of FAs and cholesterol while regulating several key lipogenic enzymes such, as ATP citrate lyase (ACLY), ACC1, and FASN, all of which support CSC stemness (Table 1) [81]. Modification in SREBP1 has been linked to increased lipogenesis, tumor proliferation, and poor prognosis in hepatocellular carcinoma (HCC) [82]. Lipid profiling in osteosarcoma patients has revealed elevated levels of cholesterol, choline, polyunsaturated fatty acids (PUFAs), and glycerol during metastasis [83]. However, the precise mechanism by which desaturase inhibitors affect CSCs remains to be fully elucidated. In this context, metabolomic and lipidomic approaches could prove valuable in studying different stages of cancer, with lipid profiling offering the potential for identifying biomarkers, monitoring cancer progression, aiding in diagnosis, and novel therapeutic targets in various cancers [83].

**Table 1:** List of Enzymes Involved in Lipid Metabolism and Dysfunction in Various Cancers.

Enzymes	Molecular and Cellular Activity	Effects in Cancer	References
Acyl-CoA synthase long-chain 3 (ACSL3)	Converts free fatty acids into fatty acyl-CoA	In ER-negative breast cancer development and progression	[84]
Acetyl-CoA carboxylase1/2	Promotes conversion of acetyl-CoA to malonyl-CoA (associated with SCD)	Support tumor growth	[85]
Acyl-CoA cholesterol acyltransferase 1 (ACAT1)	Involved in cholesterol esterification Inhibits apoptosis via increase expression of caspase 3/7 activity Decreased mitochondrial membrane potential	Leukemia, glioma, breast, pancreatic, ovarian, and prostate cancer	[86]
Cyclooxygenase 2	Involved in inflammation and tumor stroma interaction	Promotes tumor growth	[87]
Choline kinase	Required for synthesis of Phospholipids	Poor prognosis for cancer	[88,89]
Carnitine palmitoyl-transferase-1A (CPT1A) and 1B	Formation of fatty acyl-carnitine and transport of fatty acyl-carnitine and long-chain fatty acids across the inner mitochondrial membrane	Breast cancer, prostate cancer, lung cancer, and ovarian cancer metastasis	[90]
Diacylglycerol (DAG)-acyltransferase, diacylglycerol O-acyltransferase 1(DGAT1) and DGAT2	Catalyzes the esterification of fatty acid (FA)-CoA with DAG to produce triglycerides, a main component of LDs	In glioblastoma, acts as an antioxidant and prevents cell death	[91]
2, 4,-dienoyl CoA reductase (DECR1/2)	FAO of PUFAs depends on NADPH	Castrate-resistant prostate cancer (CRPC)	[92]
Fatty acid desaturases	Facilitates the conversion of saturated to unsaturated fatty acids, regulated by NF-κB	Facilitates EMT conversion	[93]
Fatty acid 2-hydroxylase (FA2H)	Hydroxylation of the C-2 position of free fatty acids	Promotes lung metastasis and invasion	
3-hydroxy-3-methylglutaryl-CoA synthase (HMGCS1/2) and 3-hydroxy-3-methylglutaryl-CoA reductase (HMGCR)	Involved in cholesterol synthesis, Acetyl-CoA ketogenesis in mitochondria	In ER-breast cancer metastasis and gastric cancer	[86]
Lipoxygenase (LOXs), ALOX15	LOX5/12/15 catalyze lipid peroxidation, mainly in a Fe^2+^—dependent manner	In gastric cancer-promoting ferroptosis	[94]
Lysosomal acid lipase (LAL)	Controls neutral lipid metabolic signaling Hydrolyzes cholesteryl ester and triglycerides in lysosomes Produces FFA and cholesterol	Role in myeloid-derived suppressor cells and tumorigenesis	[95]
Phospholipase A2	Hydrolyze the sn-2 ester bond of the phospholipids	In ovarian cancer	[96]
Stearoyl-CoA desaturase (SCD1)	Introduces a double bond at Δ^9^ position in fatty acids Produces more MUFAs; Triggers an iron-dependent cell death called ferroptosis	Poor prognosis in Stage II colon cancer, lung cancer, and endometrial cancer	[97]
Spinster homologue 2 (SPNS2)	Transporter for lipid sphingosine-1-phosphate Helps in lymphocyte trafficking	Tumor progression, lung metastasis, hepatocellular carcinoma, and poor prognosis in cancer	[98]
Sterol regulatory element binding proteins (SREBPs)	Helix-loop-helix leucine zipper transcription factors Activates storage of cholesterol and fatty acids into LDs Transcriptional regulation of lipogenesis Poor prognosis in cancer	In HCC tumorigenesis and metastasis	[99]

## 4. Cholesterol Metabolism in CSC

The presence of elevated cholesterol levels has been linked to cancer progression and poor prognosis. They are synthesized via mevalonate (MVA) pathway, which is upregulated in various cancers, including breast, lung, and HCC. Targeting this pathway has shown to decrease cell proliferation. Cholesterol biosynthesis leads to the formation of lipid rafts, which sequester proteins such as CD44, CD24, CD133, and CXCR4 associated with CSCs [91]. The MVA pathway primarily regulates the biosynthesis of steroid hormones, cholesterol, and nonsteroidal isoprenoids. Targeting key enzymes of MVA pathway, such as 3-hydroxy-3-methylglutaryl-CoA synthase (HMGCS1) and 3-hydroxy-3-methylglutaryl-CoA reductase (HMGCR), reduces cancer cell proliferation and migration [69]. Carnitine palmitoyl-transferase 1 (CPT1) assists the transfer of long-chain fatty acids into the mitochondrial membrane for oxidation, promoting acetyl-CoA and ATP production, thus promoting CSC growth [92]. In CRC metformin, an AMPK activator, along with an HMG-CoA reductase inhibitor, or a mTOR inhibitor, significantly reduces the CSC population. However, the CSC population rebounded after the addition of mevalonic acid. This suggests that mevalonic acid mitigates the inhibitory effects of these therapies on CSCs [100].

Increased cholesterol uptake through low-density lipoprotein receptors (LDLR) is associated with poor prognosis in breast and lung cancer [93]. Scavenger receptor class B type I (SR-BI) facilitates cholesterol uptake via high-density lipoproteins (HDLs). Targeting SR-BI reduces cholesterol uptake and proliferation in breast and prostate cancers [94]. Another enzyme, acyl-CoA cholesterol acyltransferase 1 (ACAT1), assists in cholesterol esterification, which is essential for cancer cell growth. Inhibition of key enzymes such as ACAT1 by avasimibe reduces tumor growth and metastasis in vivo in prostate cancer. A similar inhibitor, avasimin, induces apoptosis in various cancers, including colon, pancreatic, lung, and prostate cancer, exhibiting promising anticancer effects [95]. Liver X receptor (LXR), a nuclear hormone receptor with an important function in cholesterol metabolism, consists of two isoforms: LXRα (NR1H3) and LXRβ (NR1H2), expressed in the liver, intestine, adipose tissue, and macrophages. The LXR signaling agonist GW3965 reduces LDR receptors and increases ABC transporter A1 (ABCA1) expression, resulting in decreased intracellular cholesterol and reduced proliferation in clear cell renal cell carcinoma [96]. Atorvastatin, a lipophilic statin, has shown increased survival in HMGCR-positive breast cancer. Another drug, such as medroxyprogesterone acetate (MPA), suppresses the breast tumor spheres via inhibition of cholesterol synthesis [101]. These findings indicate that cholesterol homeostasis is important for CSC growth and survival; thus, this could be a promising target in various cancers.

## 5. Fatty Acid Oxidation in CSCs

CSCs rely on increased FAO for growth and survival and assists in drug resistance. The FAO process in CSCs utilizes acetyl-CoA and NADH to produce ATP, maintaining energy homeostasis that contributes to their resilience against treatment. Higher levels of certain enzymes, such as ATP-citrate lyase (ACLY), ACSS1, and ACSS2, have been observed in certain cancer types, promoting tumor growth and metastasis by providing an alternative energy source to glucose (Table 1) [102]. For instance, FAO supports the maintenance of stemness in mesenchymal stem cells in advance gastric cancer [103]. In pancreatic ductal adenocarcinoma (PDAC), CSC and non-CSC-targeting OXPHOS significantly impair the tumorigenicity and chemoresistance. PaCSCs, enhanced expression of lipid metabolism genes such as MGLL, PPARD, and CPT1A, have been correlated with poor prognosis with worse outcomes. Moreover, CPT1A overexpression was significantly effective in circulating PaCSCs compared to primary tumors, suggesting a survival advantage for cells with increased lipid storage and metabolism in the blood. PaCSC-enriched conditions (spheres or CD133^+^ cells) showed a significantly high LD content than non-CSCs (adherent cells or CD133^−^), suggesting that the differential distribution of lipid content is dependent on stemness. Mascaraque et al. have demonstrated that inhibition of FAO enhanced the sensitivity of PDAC cells to chemotherapy drugs, such as gemcitabine [104]. Furthermore, FAO activates the oncogenic protein Src and promotes CSC metastasis in triple-negative breast cancer (TNBC) [105]. Metastatic TNBC maintains high levels of ATP through FAO and activates the Src oncoprotein via autophosphorylation at tyrosine 419 (Y419) [105]. Moreover, leukemic stem cells (LSCs), deficient in CPT1A, the rate-limiting enzyme in FAO, exhibit resistance to avocatin B, a lipid derived from avocado that selectively targets AML stem cells with minimal impact on normal counterparts [106]. These findings underscore the critical role of FAO in the development of chemoresistance in CSCs.

Lipid peroxidation of specific fatty acids has been identified as a trigger for ferroptosis, an iron-dependent form of cell death notably observed in RAS-mutated cancer cells [107]. The excessive iron-catalyzed peroxidation of membrane phospholipids, particularly those containing the polyunsaturated fatty acid arachidonic acid (AA), plays a critical role in driving ferroptosis [108]. Phadnis et al. reported that MMD, a scaffold protein located in the Golgi apparatus, increases susceptibility to ferroptosis in ovarian and renal cancer cells through a mechanism dependent on acyl-coenzyme A (CoA) synthetase long-chain family member 4 (ACSL4) and membrane-bound O-acyltransferase family member 7 (MBOAT7) [109]. This mechanism facilitates the incorporation of AA into phosphatidylinositol (PI), thereby enhancing ferroptosis sensitivity in OVCAR-8 ovarian cancer cells [110]. However, the biochemical mechanisms underlying ferroptosis in other cancer types remain to be thoroughly investigated and compared with non-cancerous cells, as well as in in vivo models.

The AMPK pathway is crucial for energy metabolism and regulates mitochondrial FAO. AMPK inhibits certain enzymes, such as ACC1 and ACC2, leading to increased FAO and NADPH production, supporting cancer cell survival [111,112]. The AMPK/mTOR pathways have been shown to regulate lipid metabolism through different mechanisms, with AMPK promoting FAO, while mTORC1 enhances lipogenesis [113]. AMPK inhibitors, such as metformin, dorsomorphin (Compound-C), and Bay-3827, have been found to decrease FAO, potentially inhibiting cancer cell survival, although their off-target effects are a significant concern [114]. The role of AMPK/mTOR pathway in maintaining lipid metabolism in CSCs under nutritional deficiency warrants further investigation [115].

## 6. Signaling Pathways Involved in Lipid Metabolism in CSCs

**Figure 3:**
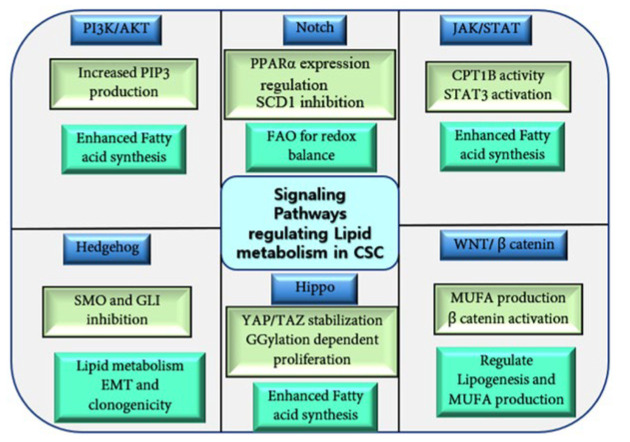
Schematic representation of the key signaling pathways involved in regulating lipid metabolism in CSCs. The figure highlights critical factors within each pathway that regulate lipid metabolism, including FAO, lipogenesis, and LD formation in cancer cells. These pathways are pivotal in controlling lipid levels, their accumulation, and metabolism, all of which contribute to cancer progression and drug resistance.

Lipid reprogramming in CSCs assists self-renewal, proliferation, and resistance to treatment [116]. Alterations in lipid metabolism lead to enhanced lipogenesis, increased free lipids content in LDs that can be easily transferred to other cells [117]. The Pi3K/AKT/mTOR, JAK/STAT, Hippo, Wnt, and NF-κB signaling pathways are essential for regulating lipid metabolism in CSCs. Activated PI3K/AKT pathway supports cell growth through increased PIP3 production in various cancers (Figure 3) [118]. In prostate cancer, the silencing of the tumor suppressor PTEN enhances the expression of enzymes involved in FA synthesis, promoting CSC metastasis and growth [119].

Notch signaling is implicated in lipid metabolism. Notch1 signaling can regulate the expression of peroxisome proliferator-activated receptor α (PPARα) and lipid oxidation genes to maintain lipid levels in hepatocyte and adipocyte cells [120]. The selective elimination of colon CSCs can be achieved by targeting Notch and Wnt signaling, specifically inhibiting SCD1 (Figure 3) [121]. Notch 1 plays a key role in utilizing FAO to maintain redox balance in quiescent endothelial cells (QECs). Supplementation with acetate, which is metabolized to acetyl-CoA, restores endothelial quiescence and counteracts oxidative stress-induced endothelial dysfunction in CPT1A-deficient mice, highlighting potential therapeutic avenues. Thus, QECs rely on FAO for vasculoprotection against oxidative stress [122].

The Hippo-YAP/TAZ signaling pathway regulates the stemness in CSCs. In breast cancer, the Hippo pathway facilitates geranylgeranylation (GGylation)-dependent cell proliferation and migration [123,124]; however, the YAP signaling pathway promotes cell proliferation, migration, and invasion in gastric cancer [124]. The YAP or TAZ also facilitates self-renewal and tumorigenesis in CSCs [125,126,127]. In lung cancer, SCD1 plays a role in maintaining fatty acid metabolism and promotes CSC stemness by stabilizing YAP/TAZ and promoting their nuclear localization (Figure 3) [128]. Further, silencing of SCD1 induced selective apoptosis of ALDH-1A1-positive cells and impaired in vivo tumorigenicity of 3D lung CSCs [129].

The JAK/STAT pathway, commonly activated in many cancers, plays a role in hematopoiesis and self-renewal of normal embryonic stem cells. Inhibition of JAK/STAT signaling reduces CSC stemness in AML [130]. Targeting JAK/STAT3 pathway via CPT1B inhibitors blocks breast CSC self-renewal and tumor growth in vivo [40].

Wnt signaling is implicated in regulating lipogenesis in CSCs [131]. In CSCs, the β-catenin pathway regulates fatty acid metabolism through YAP/TAZ signaling, particularly by modulating SCD1. In vivo studies have shown that the Wnt/β-catenin pathway is linked to lipid metabolism in mouse liver hematopoietic stem cells, where SCD-derived MUFAs establish a positive feedback loop by stabilizing and upregulating Lrp5/6 mRNA, thus enhancing Wnt signaling (Figure 3) [128]. MUFAs are essential for the synthesis and release of Wnt ligands. In CRC, Wnt-signaling dysregulation and a high-fat diet disrupt bile acid distribution, activate FXR, and drive the malignant transformation of the Lgr5+ CSC subpopulation, promoting the progression from adenomas to adenocarcinomas [132]. Additionally, the Wnt/β-catenin pathway regulates lipogenesis and MUFA production, as well as de novo adipogenesis in BCS through the upregulation of ACC, FASN, and SREBP1-c expression [133].

Moreover, various antioxidant pathways, such as Glutathione peroxidase 4 (GPX4), ferroptosis inhibitory protein 1 (FSP1), dihydroorotate dehydrogenase (DHODH), Fas-associated factor 1 (FAF1), and Tetrahydrobiopterin (BH4), act as antagonists by inhibiting ferroptosis under ROS-induced stress (Figure 3) [134,135,136,137]. The nuclear factor erythroid 2 (Nrf2), a key transcription factor in cellular antioxidant responses, upregulates ferroptosis-inhibiting molecules to prevent ferroptosis [138].

The Hh signaling pathway plays a crucial role in maintaining CSC stemness, particularly by influencing EMT in breast cancer and mammary gland morphogenesis [139]. It supports the expansion of mammary progenitors derived from mammary stem cells and regulates the differential usage of the TP63 promoter, thereby enhancing clonogenicity [140]. Cyclopamine, an inhibitor of the transmembrane protein SMO, has shown efficacy against CML stem cells in vitro and in vivo [141], while GANT61, a GLI, inhibitor, blocks GLI function, inhibiting tumor cell growth (Figure 3). GANT61 has demonstrated potent effects in inducing cell death in colon carcinoma, neuroblastoma, and chronic lymphocytic leukemia [141,142,143]. GLI1 expression is upregulated in CD34+ subpopulation of AML cells. GANT61 induces AML cells’ apoptosis and differentiation. The combination of GANT61 with chemotherapeutics has shown a synergistic anti-proliferative effect on primary CD34+ AML cells [144]. These findings highlight the intricate connection between Hh signaling, lipid metabolism, and CSC regulation, suggesting potential therapeutic targets for cancer treatment.

## 7. Autophagy as an Essential Player in Maintaining CSC Stemness

**Figure 4:**
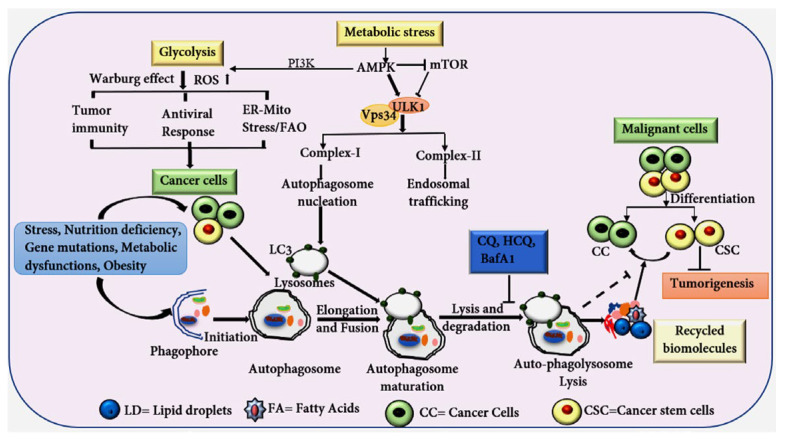
The diagram shows the process of autophagy and its critical role in regulating the stemness of CSC under various stress conditions, such as hypoxia, nutrient deprivation, increased FAO, elevated ROS, therapeutic interventions, and autophagy activation. Autophagy facilitates the removal of damaged organelles, like mitochondria, protein aggregates, and misfolded proteins and lipids. The degradation products are recycled to provide essential nutrients, which support both CCs and CSCs, preserving their stemness. Inhibition of autophagy at either early or late stages significantly impacts the CSC fate, potentially impairing their stemness and survival. Glycolysis and AMPK signaling also support CSC stemness under stress conditions. These pathways support tumor microenvironment (TME), promoting resilience and therapeutic resistance in CSCs.

Autophagy is a lysosomal-dependent mechanism crucial for metabolic recycling and cell survival under conditions of stress and nutrient deprivation [145]. This process involves various steps: initiation, autophagosome formation, elongation, and, finally, lysosomal degradation [146]. Autophagy plays a vital role in maintaining intracellular homeostasis by removing damaged organelles, misfolded or damaged proteins, recycling them to sustain cellular homeostasis. This mechanism is intricately associated with the preservation of CSC stemness and consequently facilitates tumorigenesis and drug resistance [147,148]. CSCs often exhibit elevated levels of autophagy, as indicated by the increased expression of autophagy-related genes, such as ATG4, ATG5, and Becline1, displaying an elevated autophagic flux (Figure 4) [149]. Interestingly, while autophagy supports tumor growth by maintaining CSC viability, it also acts as a tumor suppressor by destabilizing the transcription factor NRF2, thus helping tumor cells resist oxidative stress [150].

The role of autophagy in CSC proliferation and tumorigenesis is context-dependent, involving processes such as lipophagy, where LDs merge with autophagosomes, and increased FAO, which further amplifies the growth-promoting effects in CSCs [151]. Forkhead box 3A (FOXA3), a transcription factor, induces the expression of autophagy-related genes, such as LC3, ULK1, ATG5, and GABARAPL1 and beclin-1 in stem cells, and has been implicated in regulating CSC fate (Figure 4). The loss of FOX3A has been shown to enhance CSC stemness in various cancers, such as glioblastoma, ovarian, breast, liver, and colorectal [152,153,154]. Moreover, the knockdown of ATG5 in ovarian cancer stem cells reduces chemoresistance and self-renewal capacity (Figure 4) [155]. Moreover, the basal level of autophagy/mitophagy is higher in BCSCs compared to normal tissue stem cells, and autophagy induced the upregulation of CD44 and vimentin, both of which are recognized as stem cell markers [156].

Mitophagy, a specialized form of autophagy that targets damaged mitochondria for degradation, is implicated in CSC maintenance. Elevated ROS levels can impair normal mitochondrial functions, leading to cell death. However, CSCs possess enhanced anti-oxidative capacity, which protects them from ROS-mediated cell death. In hematopoietic stem cells (HSCs), the knockdown of PINK1/parkin suppresses mitophagy and reduces stemness [157]. CSCs leverage elevated mitophagy to regulate ROS and FAO, thus supporting their survival and proliferation [158].

Autophagy inhibitors have shown efficacy in sensitizing resistant tumor cells to chemotherapy and radiotherapy. For instance, drugs such as clomipramine and chloroquine, have been demonstrated to induce cell death in enzalutamide (ENZA)-resistant prostate cancer cells [159]. The combination of standard cancer therapies with autophagy inhibitors has proven to be more effective than either approach alone in targeting CSCs [160]. In GSCs, combining paclitaxel (PTX) and chloroquine phosphate (CQ) enhanced the efficacy of chemotherapy [161]. Furthermore, combining autophagy inhibitors like CQ and hydroxychloroquine (HCQ) with other cancer drugs has been shown to improve treatment outcomes, as evidenced in gastrointestinal stromal tumors where the combination of CQ and imatinib promotes apoptosis [149,162].

## 8. Lipid Metabolism and Autophagy Pathway Crosstalk in CSC

The metabolic reprogramming within TME is influenced by multiple factors, including enhanced FAO, heightened lipid uptake, and elevated expression of FASN. These alterations result in higher levels of lipids and fatty acids (FAs), leading to the induced accumulation of LDs in cancer cells. In CSCs, FAs are stored in LDs as triacylglycerols (TAGs) and sterol esters, maintaining lipid homeostasis and preventing lipotoxicity [54]. Autophagy plays a pivotal role in regulating the amount of lipids and FAs in CSCs by breaking down LDs and releasing FAs through lipophagy (Figure 4). This phenomenon has been observed in various cancers and can suppress tumor growth through mechanisms such as ferroptosis, ROS production, and ER stress [163].

Autophagy also supports CSC stemness by providing essential nutrients, particularly under stress conditions, such as hypoxia, nutrient deprivation, energy ablation, and exposure to chemotherapy or radiation [115]. Under starvation conditions, TAGs stored in cytoplasmic LDs are degraded through lysosomes-mediated autophagy, a process driven by patatin-like phospholipase domain-containing protein 2 (PNPLA2). This breakdown releases free FAs, which can either be transported to the mitochondria for β-oxidation or re-esterified into LDs [164]. Additionally, a deficiency in glucose-6-phosphatase (G6P), whether induced genetically or chemically, has been shown to trigger autophagy, reducing lipid accumulation and liver steatosis. This suggests that G6P could be a promising target for reducing lipid buildup and limiting tumorigenesis in cancer cells [165].

Lysosomal acid lipase (LAL) is essential for autophagy and lipid degradation. Inhibition of LAL has been linked to hematopoietic abnormalities and the suppression of immature myeloid-derived suppressor cells (MDSCs), which can impair immune surveillance and promote tumorigenesis [95,166].

## 9. Targeting Lipid Metabolism and Autophagy Pathway in CSCs

**Figure 5:**
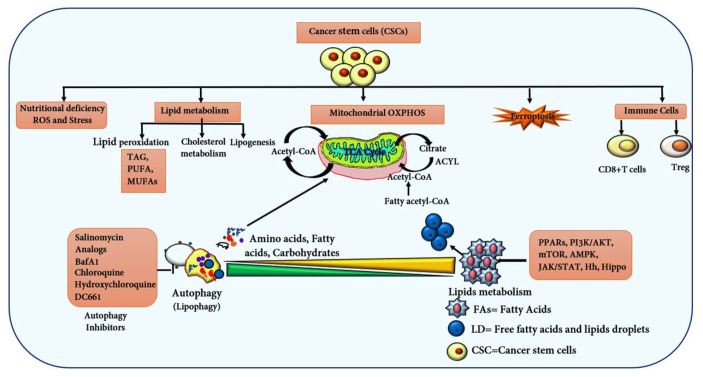
The diagram illustrates the connection between autophagy and lipid metabolism in CSCs, highlighting the central role of mitochondria in sustaining CSCs through metabolites from fatty acid metabolism and autophagy degradation. Key enzymes facilitate the production of Acetyl CoA, crucial for FAO, the TCA cycle, and ATP generation. The diagram also depicts lipid peroxidation and the role of GPX4 in inducing ferroptosis in CSCs. Additionally, CD8+ and Treg cells are shown to regulate CSC stemness. Various inhibitors targeting autophagy, lipid metabolism, and FAO at critical regulatory points are included, aiming to eliminate CSCs.

Cancer therapies targeting the TME through lipid metabolism, FAO, and degradation pathways, such as lipophagy and ferroptosis, offer promising approaches to inhibit tumorigenesis (Figure 5). Modulating these pathways can alter lipid uptake in cancer cells, including CSCs, significantly affecting cancer progression. Inhibition of key enzymes like ACC and FASN has shown considerable efficacy in eliminating CSCs. Targeting ACC in pancreatic cancer cells has been shown to effectively inhibit tumor proliferation both in vivo and in vitro, which is mediated through the reduction of palmitoylation key ligands involved in the Wnt and Hh signaling pathways, which are critical for maintaining CSC characteristics [167]. Inhibiting FASN in HER2-positive advanced breast cancer cells has reduced cancer growth in both preclinical and clinical studies. Soraphen A, an inhibitor, has shown a potential effect on breast CSCs and has proven effective in NSCLC and HCC cancers (Table 2) [61,168,169]. Targeting CPT1A, a critical enzyme in FAO, with inhibitors such as etomoxir and ranolazine, has been shown to reduce fatty acid transport into mitochondria, thereby suppressing tumor growth [170]. Napabucasin (BBI608) has demonstrated significant efficacy in metastatic colorectal and pancreatic cancer [171]. Additionally, targeting ALDH+ breast CSCs with disulfiram and gemcitabine has led to tumor growth inhibition and enhanced T-cell mediated immunity [172]. Fresolimumab, a TGF-β blocker, has improved immune response and extended survival in clinical studies involving breast cancer patients [173]. ALDH inhibitors, such as disulfiram and its derivatives, have also enhanced chemotherapy sensitivity in lung cancer [174].

Breast CSCs exhibit increased levels of long-chain FAO metabolites, and treatment with etomoxir has successfully inhibited FAO, reducing stem cell viability and tumor sphere formation [40]. Furthermore, inhibitors targeting the Hh signaling pathway, such as Sonidegib (LDE225) and Glasdegib (PF-04449913), have shown long-term survival benefits in patients with basal cell carcinoma (BCC) and acute myeloid leukemia (AML), respectively [175,176]. The enzyme HMGCR, a key enzyme for cholesterol biosynthesis, upregulated in gastric cancer, activates the Hh/Gli1 signaling pathway. Targeting Hh signaling with statins in combination with cyclopamine has effectively suppressed gastric cancer progression [177].

Inhibition of ACYL in CSCs has not shown significant effects due to the compensatory role of ACSS2, which replenishes acetyl-CoA in the absence of ACLY in cancer models [178]. In vitro inhibition of ACYL in A2780/CDDP ovarian cancer (OC) cells reduced cisplatin resistance by attenuating the PI3K-AKT pathway and activating the AMPK-ROS signaling. These findings suggest that combination of an ACYL inhibitor with cisplatin might represent a promising therapeutic strategy for overcoming cisplatin resistance in OC [179]. Additionally, dephosphorylation and inactivation by PI3K inhibitors have not yielded substantial results in lung cancer treatment [180]. Conversely, inhibiting SCD1 in CSCs has shown promise in impairing cell proliferation, particularly in normal human fibroblasts [181]. The SCD1 inhibitor MK-8245 has demonstrated significant effects in Phase II clinical trials in liver disease [182]. Betulinic acid (BetA) has been identified as a potential inhibitor of SCD in CRC cell lines such as HCT116, SW480, and DLD-1, exhibiting time-dependent antiproliferative effects. In vivo, BetA suppressed tumor growth in a HCT-116 xenograft tumor mouse model [183]. Additionally, BetA induced rapid cell death in CSCs by eliminating their clonogenic capacity [184]. Elevated SCD1 levels, associated with increased MUFAs, have been observed in various cancers, such as lung, ovarian, breast, and GSCs [185]. SCD1 has been shown to regulate the Wnt signaling in CSCs and play a crucial role in CSC maintenance in various cancers, including HCC and CC [15,186,187]. In glioblastoma, Kloosterman et al. demonstrated that lipid-laden macrophages (LLMs), a type of brain-resident microglia that scavenge myelin-derived lipid debris, promote tumor aggressiveness [188]. Inhibiting lipid uptake with the CD36 inhibitor sulfosuccinimidyloleate (SSO) or ABCA1/LXR inhibitors reduced LLM-induced proliferation in mouse glioblastoma models [188,189]. These findings suggest that targeting lipid uptake in CSCs may be a promising therapeutic approach to reduce stemness across various cancers.

Autophagy plays a crucial role in cancer progression and metastasis, as indicated by the elevated autophagy markers expression in CSCs across various cancers. including gastric, colorectal, ovarian, and liver [190]. Numerous small-molecule inhibitors have been developed to target different stages of the autophagy. For example, ULK1, a key regulator of the initiation phase of autophagy, has emerged as a promising target in multiple cancers. Specific inhibitors, such as ULK-101, have demonstrated efficacy in inhibiting autophagy in KRAS-driven lung cancer cells under nutrient deprivation [191]. Other ULK1 inhibitors, such as MRT67307, MRT68921, and SBI-0206965, have shown potential in preclinical studies (Table 2) [192,193]. Inhibitors targeting Vps34, such as 3-MA, wortmannin, LY294002, spautin-1, SAR405, autophinib, vps34-IN1, and PIK-III, require further optimization for dosage and specificity across various cancers [194]. ATG4B, which is highly expressed in cancer and contributes to metastasis and drug resistance, is considered a potential anticancer target. Inhibitors such as Z-FA-FMK, FMK-9a, NSC185058, and S130 have shown significant inhibition of ATG4B, with promising results in preclinical cancer models (Table 2). Late-stage autophagic inhibitors like CQ and HCQ have shown notable effects on cancer cells in vitro and in vivo. New derivatives, such as DC661, have demonstrated significant inhibition of autophagy in specific cancer cell lines, even at low concentrations [195]. Palmitoyl-protein thioesterase 1 (PPT1), a molecular target of HCQ, Lys05, and DC661, is overexpressed in various cancers and is associated with poor prognosis [196]. DC661 has also enhanced the sensitivity of HCC cells to sorafenib [196].

Additionally, inhibitors targeting V-ATPase, which is essential for autophagy and lysosomal acidification, have shown potential in enhancing the anticancer effects of various compounds [197]. For instance, bafilomycin A1 (BafA1) has been found to enhance the efficacy of tyrosine kinase inhibitor (TKI), such as imatinib mesylate, in various cancers [198]. Natural compounds like toosendanin (TSN) and ginsenoside Ro have also demonstrated the ability to inhibit V-ATPase. Notably, ginsenoside Ro has sensitized chemoresistant esophageal cancer cells to 5-fluorouracil (5FU) [199]. Although further investigation is required to elucidate the mechanisms and specificity to these inhibitors across various cancers, their promising effects in combination with anticancer drugs warrant additional validation for potential for clinical applications.

**Table 2:** List of Small Molecules, Cancer Drugs, Autophagy, and Lipid Metabolism Inhibitors in CSCs.

Chemicals, Inhibitors and Drugs	Targeted Molecules and Pathways	Molecular Mechanism	Cancer Types	References
A-922500	Inhibits DGAT1	Reduces LDs Increases cancer cell death	Prostate cancer	[200]
Avasimibe, Avasimin	Inhibits Acyl-CoA cholesterol acyltransferase 1	Increase apoptosis	Colon, prostate, lung, and pancreatic cancer	[201]
AZ22, AZ65	FASN inhibitor	Inhibit FASN-mediated cell growth in cancer	Breast cancer (reduces tumor growth)	[180]
Benzothiazoles and oxalomides, A-939572, CAY10566, CVT-11127	Targets stearoyl-CoA desaturase (SCD)	Inhibits SCD and induces cell cycle arrest	NSCLC, colon, thyroid, and glioblastoma	[202]
BMS309403 (Biphenyl azole compound), BD62694	Inhibits fatty acid binding protein 3 and 4 (FABP3, FABP4)	Reduces lipid accumulation Reduces cell cycle genes (CycD1, VEGFA, and VEGFR)	Prostate cancer, colon cancer, ovarian cancer, and lung metastasis	[203,204]
Cerulenin, C75, C93	Targets FASN targeting CPT1	Reduces stemness markers SOX2, CD133, and FABP7 Induces apoptosis	Glioma stem cells, oesophageal, and squamous cell carcinoma	[205,206]
CAY 10566, SC-26196	Represses NF-κβ activation Promotes AMPK activity and lipophagy	Inhibits SCD1 activity	Induces hepatic steatosis in HSCs	[207]
Cerivastatin	Hydroxy-methylglutaryl-CoA reductase	Inhibits the mevalonate pathway	Breast tumors	[208]
Carbamazepine	Effects on KRAS mutant Prevent steatosis	Effective on early-stage autophagy via ULK1	HCC and colorectal cancer	[209]
Clomipramine, Chloroquine	Induces the expression of PUMA	Defective mitochondrial function	Breast cancer	[194]
Cabozantinib	Inhibits multi-tyrosine kinase	Inhibits VEGF, MET, and AXL	Metastasis renal cell carcinoma, metastatic medullary thyroid cancer, and HCC	[210]
Crizotinib	Inhibits multi-tyrosine kinase	Targets ROS1, EML4, and ALK gene alterations	Non-small cell lung cancer (NSCLC), renal cell carcinoma, HCC, and thyroid cancer	[211,212]
Disulfiram and gemcitabine, etomoxir	Targets aldehyde dehydrogenase ALDH+ breast cancer stem cells	Enhances T-cell immunity Promotes tumorigenesis and drug resistance	Breast cancer and lung cancer	[64,172]
Dacarbazine (DTIC)	Effect on diet-induced obesity and DNA repair	Hyperthermia potentiated its effect in melanoma cell lines	Tumor-bearing HFD-fed mice and Hodgkin lymphoma	[213]
Etomoxir, Perhexiline, ST1326	Inhibits carnitine palmitoyl transferase 1 (CPT1)	Reduces ATP level; Decreases cell viability, inducing apoptosis	BCSC, HCC, and colorectal cancer	[40,90]
Fresolimumab	Blocks the activity of TGF-β isoforms	Shows tumor suppressor response	Breast cancer and renal cell carcinoma	[173,214]
GW3965, LXR623	Liver X receptor (LXR) agonist Agonist of LDR receptors Induced ABC reporter (ABCA1)	Reduces intracellular cholesterol effects on proliferation and tumorigenesis	Clear cell renal cell carcinoma	[215]
GSK165	Inhibits ACLY activity in concentration-dependent manner	Antiproliferative effect on HT29 CRC cells	In CRC-induced sensitivity to anti-neoplastic drug SN38	[216]
Lipofermata and arylpiperazine 5K (DS22420314)	Inhibits the FATP1	Inhibits the uptake of long-chain fatty acids	In ER+ breast cancer	[217]
Metformin and statins	Targets lipid synthesis HMGCR via AMPK-mTOR	Poor prognosis with elevated PLIN1 and DGAT1	Prostate cancer	[200]
2-Methylthio-1,4-naphthoquinone (MTN)	Suppresses lipid uptake and reduces the CSC population	Inhibits CD36 ligands Induced apoptosis in CSC via Caspase 3/7 levels	Glioblastoma multiforme	[218]
ND-646, ND-630, 5-Tetracepoxy-2-furan Acid (TOFA)	Targets ACC1/2	Inhibits FAS Activates FAO	NSCLC lung cancer and HCC	[219]
Orlistat, TVB-2640, GSK2194069	Inhibits FASN activity	Inhibits lipase	Breast, colon, and ovarian cancer	[220,221]
Oligomycin A, antimycin A	Inhibits autophagy	Reduces CSC numbers Induces cytotoxicity	Glioblastoma stem cells	[222]
Resveratrol (3,5,4′-trihydroxystilbene)	Inhibits FASN	Binds to ketoacyl reductase domain	CRC cell proliferation and elevates the apoptosis	[223]
Salinomycin	Acts as ionophore Induces ROS and apoptosis Inhibits lysosomal activity and autophagic flux	Inhibits p-glycoprotein efflux pump Induces apoptosis via reducing CDKN1A/p21 level	ALDH+ cancer cells and BCSCs	[224]
Soraphen A, ND654, 5-tetracepoxy-2-furan acid (TOFA)	Inhibits Acetyl-CoA carboxylase and SCD1	Inhibits ACC catalytic activity	Breast cancer, lung cancer, and prostate cancer	[61,168,169]
Statins, fluvastatin, lovastatin	Inhibits farnesyl pyrophosphate (FPP) and geranylgeranyl pyrophosphate (GGPP) HMG-CoA reductase	Reduces cancer cell proliferation and migration	Prostate, lung, and ovarian cancer	[61]
SSI-4, MF-438, betulinic acid (BetA)	Inhibits Stearoyl-CoA desaturase 1 (SCD)	Induces apoptosis in cancer cells via modulating mitochondrial dynamics, ER stress	HCC, colorectal cancer, and lung cancer	[54]
Simvastatin, LY2157299 (galunisertib)	Inhibits HMG-CoA reductase and EMT antagonist via TGF-β pathway	Reduces vimentin level, β-catenin Inhibits migration and invasion	Bladder cancer, glioblastoma, rectal cancer, pancreatic cancer, HCC, and lung cancer	[13,225]
Sulfosuccinimidyl oleate (SSO)	Targets CD36	Inhibits cancer stem cell growth Reduces migration of CCs	Ovarian cancer and HCC	[226]
5-(tetradecyloxy)-2-furancarboxylic acid (TOFA)	Inhibits ACC activity	Induces apoptosis in dose-dependent manner	In CRC cells HCT-8 and HCT-15	[227]
Vatalanib	Multi-targeted tyrosine kinase, an agonist for VEGFR, PDGFR, and cKit	Drug-resistant cancer cells	Colon cancer	[228]
VY-3-135, Rosiglitazone, 1-(2,3-di (thophen-2-yl) quinoxaline-6-yl)-3-(2-methoxyethyl) urea	Inhibits ACSS2 and ACSL4 activity	PPAR-γ agonist Enhances sensitivity to chemotherapeutic drugs	Breast and bladder cancer	[229]
Vorinostat	Inhibits histone deacetylase	Induces apoptosis and autophagy	Lymphomas, leukemia, and solid tumors	[230]

## 10. Discussion

CSCs pose a significant challenge in various cancer treatments, including chemotherapy, radiotherapy, and immunotherapy, due to their drug resistance. Metabolic reprogramming and autophagy play crucial roles in CSC adaptive responses, making them promising targets to overcome treatment resistance. Notably, targeting lipid metabolism and autophagy may provide therapeutic avenues for eradicating CSCs and enhancing their sensitivity to conventional treatments. CSCs exhibit elevated lipid storage compared to normal cells, promoting resistance to cancer therapy. This lipid accumulation induces FAO and modulates autophagy (Figure 5). Inhibition of key lipid metabolism enzymes and autophagy inhibitors enhances the effectiveness of cancer therapies. For instance, inhibiting FASN sensitizes breast cancer cells to doxorubicin by disrupting lipid synthesis [231]. Similarly, targeting fatty acyl-CoA synthetase (FCS) increases colon cancer cell sensitivity to 5-FU and oxaliplatin by preventing LD accumulation [232]. Moreover, inhibiting glucosylceramide synthase (GCS) in combination with chemotherapy agents, such as paclitaxel (PTX), hydroxyprogesterone (HPR), and irinotecan, has demonstrated synergistic anticancer effects [233]. Targeting fatty acid transport protein 2 (FATP2) in myeloid-derived suppressor cells (MDSCs) reduces fatty acid accumulation, enhances mitochondrial function, and reduces ROS activation, transforming MDSCs into an immune-stimulatory phenotype. This modulation enhances the efficacy of anti-PD-l cancer immunotherapy [234,235]. Additionally, inhibiting CPT1A, a key enzyme in FAO, improves antigen-specific CD8+ T-cell responses and dendritic cell (DC)-mediated T-cell priming, enhancing anti-PD-1 therapy in BRAF^V600E^ melanoma [236]. Combining lipid metabolism modulator with anti-PD-1 therapy and mRNA cancer vaccines could represent a novel and effective regimen for melanoma treatment.

Autophagy, a crucial process in CSC maintenance and survival, is tightly regulated by key molecules. Phosphorylation of ULK1 initiates autophagy by forming a complex with ATG13, FIP200, and other proteins [237,238]. Inhibiting ULK1 has shown promise in sensitizing CSCs to chemotherapy, as evidenced by the ULK1 inhibitor SBI-0206965, which, in combination with mTOR inhibitor, increases cisplatin sensitivity in non-small cell lung cancer [239]. Resveratrol, a natural compound, also inhibits mTORC1 and ULK1, preventing LC3 accumulation and inducing autophagy via AMPK signaling [240]. Deletion of autophagy-related proteins, such as ATG4A, LC3B, and ATG12, has been associated with reduced cancer cell populations and decreased expression of vimentin, a promising marker of cancer progression [156].

Several signaling pathways, including Notch in gastric cancer, JNK/STAT3 in hepatocytes, and EGFR in oral cancer, regulate autophagy and support CSC survival (Figure 5). This highlights autophagy inhibition as a potential therapeutic approach to eliminate CSCs. Inhibitors, such as CQ and HCQ, are under investigation for their ability to reduce autophagy and CSC proliferation in HCC and colorectal cancer. Additional inhibitors, including BafA1 (vacuolar H^+^ ATPase inhibitor), concanamycin A, dimeric-quinapine (DQ661), Lys05 (CQ analog), protease inhibitor E64d, V-ATPase inhibitor gastrin A, 3-methyladenine, and GNS561 (palmitoyl protein thioesterase-1) [241], along with ammonium chloride, methylamine, and siramesine (sigma-2 receptor ligand) [242,243], are undergoing clinical evaluation to determine their efficacy and optimal dosing.

Given the context-dependent nature of autophagy in cancer, which is influenced by tumor type, stage, microenvironment, and genetic factors, a comprehensive evaluation of combination drug approaches is essential. Tailoring therapeutic strategies based on cancer-specific characteristics will aid in the eradication of CSCs, inhibition of their proliferation, and improvement of treatment responses, including chemotherapy, radiotherapy, and immunotherapy.

## 11. Conclusions and Future Perspectives

**Figure 6:**
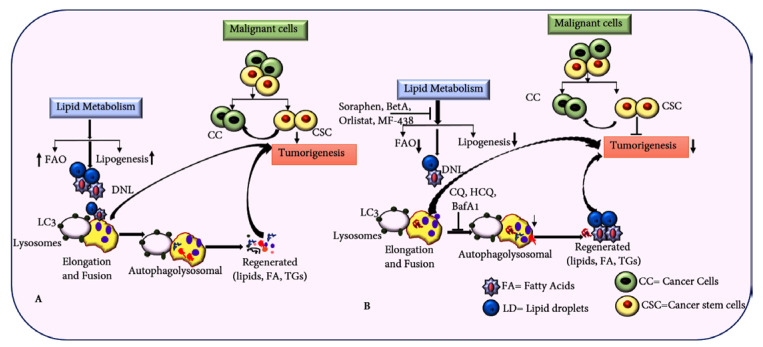
Integrated strategy targeting lipid metabolism and autophagy pathways to eliminate CSCs. (**A**) Illustration depicting how upregulated lipid metabolism supports CSC growth and proliferation. (**B**) Targeting lipid metabolism and autophagy pathways in CC can potentially inhibit tumorigenesis and metastasis driven by CSCs.

CSCs are key drivers of recurrence and treatment resistance, sustained by factors like the TME, autophagy, and immune interactions. Targeting CSCs is challenging but crucial for effective cancer eradication. Understanding the mechanisms that assist CSCs, such as quiescent stage, surface markers, CD 133, CD44v6, MET factor receptors (c-MET), epithelial cell adhesion molecules (EpCAM, CD47), and metabolic dependencies, is crucial for developing successful long-term treatments [210]. Metabolic reprogramming, especially targeting lipid and fatty acid metabolism, shows promise in overcoming CSC-mediated resistance [244]. Inhibiting key modulators involved in lipid metabolism and autophagy, such as PNPLA2, FOXO1, LIPA, ULK1, and Vps34, can enhance chemotherapy by increasing CSC sensitivity to cancer therapies (Figure 6).

This review emphasizes targeting lipid metabolism and lipophagy in CSCs. Understanding CSC metabolic dependencies, including FAO, lipogenesis, and LD accumulation, could reveal novel therapeutic targets to overcome drug resistance and prevent tumor relapse. Furthermore, combinations of chemotherapeutic drugs with inhibitors of lipogenesis, lipid uptake, FAO, and autophagy may improve cancer therapy and influence clinical trial outcomes.

## 12. Limitations and Challenges

Targeting lipid metabolism in CSCs is crucial for successful treatment. Not all the CSCs rely on lipid metabolism due to highly heterogenous and often shift to alternative energy sources like glucose, OXPHOS, FAO, and amino acids. This metabolic flexibility makes targeting lipid metabolism alone insufficient for eradicating CSCs. Additionally, multiple lipid pathways are interconnected, so targeting a single pathway may have limited efficacy, highlighting the potential need for combination therapies, which require further research. The risk of off-target effects on normal stem cells and non-cancerous tissues complicates treatment strategies. Thus, identifying key targets in lipid metabolism in CSCs, with minimum off-target effects, remains a critical hurdle.

Furthermore, the enhanced expression of ABC transporters in CSCs leads to drug efflux, reducing the effectiveness of lipid-targeting therapies. The variability in lipid sources, including FAO, de novo lipogenesis, and cholesterol metabolism, across different cancer types adds complexity. In summary, while targeting CSC lipid metabolism holds potential, addressing CSC heterogeneity, metabolic plasticity, and minimizing toxicity to normal cells are major challenges. A deeper understanding of the interplay between CSC lipid metabolism and other metabolic pathways will be crucial for overcoming these obstacles and improving therapeutic outcomes.

## Data Availability

All the references are cited in the manuscript; however, we apologize for the omission of any primary citations.

## Abbreviations

ALDH1A1	Acetaldehyde dehydrogenase 1 A1
ACAT1	Acyl-CoA cholesterol acyltransferase 1
ACLY	ATP citrate lyase
CSCs	Cancer stem cells
CPT1A	Carnitine palmitoyl transferase 1A
EMT	Epithelial-to-mesenchymal cell transition
EpCAM	Epithelial cell adhesion molecules
FAO	Fatty acid β-oxidation
FASN	Fatty acid synthase
FCS	Fatty acyl-CoA synthetase
FATP2	Fatty acid transport protein 2
FSP1	Ferroptosis inhibitory protein 1
GPX4	Glutathione peroxidase 4
G6P	Glucose-6-phosphatase
HDLs	High-density lipoproteins
LAL	Lysosomal acid lipase
LDs	Lipid droplets
LDLR	Low-density lipoprotein receptors
LXR	Liver X receptor
MDSC	Myeloid-derived suppressor cells
PPT1	Palmitoyl-protein thioesterase 1
PPARγ	Peroxisome proliferator-activated receptor γ
TME	Tumor microenvironment
TNBCs	Triple-negative breast cancer
TNF-α	Tumor necrosis factor-α
TICs	Tumor-initiating cells
TCA	Tricarboxylic acid cycle
TAN	Tumor-associated neutrophils
TAM	Tumor-associated macrophages
TAGs	Triacylglycerols
SCD1	Stearoyl-COA desaturase 1

## References

[1] Siegel R.L., Giaquinto A.N., Jemal A. (2024). Cancer statistics, 2024. CA A Cancer J. Clin..

[2] Liberti M.V., Locasale J.W. (2016). The warburg effect: How does it benefit cancer cells?. Trends Biochem. Sci..

[3] Missiroli S., Perrone M., Genovese I., Pinton P., Giorgi C. (2020). Cancer metabolism and mitochondria: Finding novel mechanisms to fight tumours. EBioMedicine.

[4] Rysman E., Brusselmans K., Scheys K., Timmermans L., Derua R., Munck S., Van Veldhoven P.P., Waltregny D., Daniëls V.W., Machiels J. (2010). De novo lipogenesis protects cancer cells from free radicals and chemotherapeutics by promoting membrane lipid saturation. Cancer Res..

[5] Chu X., Tian W., Ning J., Xiao G., Zhou Y., Wang Z., Zhai Z., Tanzhu G., Yang J., Zhou R. (2024). Cancer stem cells: Advances in knowledge and implications for cancer therapy. Signal Transduct. Target. Ther..

[6] Bonnet D., Dick J.E. (1997). Human acute myeloid leukemia is organized as a hierarchy that originates from a primitive hematopoietic cell. Nat. Med..

[7] Clarke M.F. (2019). Clinical and therapeutic implications of cancer stem cells. Reply. N. Engl. J. Med..

[8] Lacina L., Plzak J., Kodet O., Szabo P., Chovanec M., Dvorankova B., Smetana K. (2015). Cancer microenvironment: What can we learn from the stem cell niche. Int. J. Mol. Sci..

[9] Takakura N. (2012). Formation and regulation of the cancer stem cell niche. Cancer Sci..

[10] Paul R., Dorsey J.F., Fan Y. (2022). Cell plasticity, senescence, and quiescence in cancer stem cells: Biological and therapeutic implications. Pharmacol. Ther..

[11] Oskarsson T., Batlle E., Massagué J. (2014). Metastatic stem cells: Sources, niches, and vital pathways. Cell Stem Cell.

[12] Plaks V., Kong N., Werb Z. (2015). The cancer stem cell niche: How essential is the niche in regulating stemness of tumor cells?. Cell Stem Cell.

[13] Ramesh V., Brabletz T., Ceppi P. (2020). Targeting emt in cancer with repurposed metabolic inhibitors. Trends Cancer.

[14] Zhang P., Sun Y., Ma L. (2015). Zeb1: At the crossroads of epithelial-mesenchymal transition, metastasis and therapy resistance. Cell Cycle.

[15] Ma X.-L., Sun Y.-F., Wang B.-L., Shen M.-N., Zhou Y., Chen J.-W., Hu B., Gong Z.-J., Zhang X., Cao Y. (2019). Sphere-forming culture enriches liver cancer stem cells and reveals stearoyl-coa desaturase 1 as a potential therapeutic target. BMC Cancer.

[16] Zhou H., Jiang Y., Huang Y., Zhong M., Qin D., Xie C., Pan G., Tan J., Deng M., Zhao H. (2023). Therapeutic inhibition of pparα-hif1α-pgk1 signaling targets leukemia stem and progenitor cells in acute myeloid leukemia. Cancer Lett..

[17] Chen S.Z., Ling Y., Yu L.X., Song Y.T., Chen X.F., Cao Q.Q., Yu H., Chen C., Tang J.J., Fan Z.C. (2021). 4-phenylbutyric acid promotes hepatocellular carcinoma via initiating cancer stem cells through activation of ppar-α. Clin. Transl. Med..

[18] Wang Y., Wang J., Li X., Xiong X., Wang J., Zhou Z., Zhu X., Gu Y., Dominissini D., He L. (2021). N 1-methyladenosine methylation in trna drives liver tumourigenesis by regulating cholesterol metabolism. Nat. Commun..

[19] Wang DingZhi W.D., Fu LingChen F.L., Wei Jie W.J., Xiong Ying X.Y., Dubois R. (2019). Pparδ mediates the effect of dietary fat in promoting colorectal cancer metastasis. Cancer Res..

[20] Chen C.-L., Kumar D.B.U., Punj V., Xu J., Sher L., Tahara S.M., Hess S., Machida K. (2016). Nanog metabolically reprograms tumor-initiating stem-like cells through tumorigenic changes in oxidative phosphorylation and fatty acid metabolism. Cell Metab..

[21] Clancy H., Pruski M., Lang B., Ching J., McCaig C.D. (2021). Glioblastoma cell migration is directed by electrical signals. Exp. Cell Res..

[22] Basu-Roy U., Han E., Rattanakorn K., Gadi A., Verma N., Maurizi G., Gunaratne P.H., Coarfa C., Kennedy O.D., Garabedian M.J. (2016). Pparγ agonists promote differentiation of cancer stem cells by restraining yap transcriptional activity. Oncotarget.

[23] Lang C.M., Chan C.K., Veltri A., Lien W.-H. (2019). Wnt signaling pathways in keratinocyte carcinomas. Cancers.

[24] Kim J., Choi K.-W., Lee J., Lee J., Lee S., Sun R., Kim J. (2021). Wnt/β-catenin signaling inhibitors suppress the tumor-initiating properties of a cd44+ cd133+ subpopulation of caco-2 cells. Int. J. Biol. Sci..

[25] Husain K., Coppola D., Yang C.S., Malafa M.P. (2021). Farnesyl dimethyl chromanol targets colon cancer stem cells and prevents colorectal cancer metastasis. Sci. Rep..

[26] Li Z., Yang Z., Liu W., Zhu W., Yin L., Han Z., Xian Y., Wen J., Tang H., Lin X. (2023). Disheveled3 enhanced emt and cancer stem-like cells properties via wnt/β-catenin/c-myc/sox2 pathway in colorectal cancer. J. Transl. Med..

[27] Kwon J.-W., Seok S.-H., Kim S., An H.-W., Choudhury A.D., Woo S.-H., Oh J.-S., Kim J.K., Voon D.C., Kim D.-Y. (2023). A synergistic partnership between il-33/st2 and wnt pathway through bcl-xl drives gastric cancer stemness and metastasis. Oncogene.

[28] Zhang Z., Xu Y. (2022). Fzd7 accelerates hepatic metastases in pancreatic cancer by strengthening emt and stemness associated with tgf-β/smad3 signaling. Mol. Med..

[29] Qin T., Li B., Feng X., Fan S., Liu L., Liu D., Mao J., Lu Y., Yang J., Yu X. (2018). Abnormally elevated usp37 expression in breast cancer stem cells regulates stemness, epithelial-mesenchymal transition and cisplatin sensitivity. J. Exp. Clin. Cancer Res..

[30] Choi S., Yu J., Park A., Dubon M.J., Do J., Kim Y., Nam D., Noh J., Park K.-S. (2019). Bmp-4 enhances epithelial mesenchymal transition and cancer stem cell properties of breast cancer cells via notch signaling. Sci. Rep..

[31] Xiao W., Gao Z., Duan Y., Yuan W., Ke Y. (2017). Notch signaling plays a crucial role in cancer stem-like cells maintaining stemness and mediating chemotaxis in renal cell carcinoma. J. Exp. Clin. Cancer Res..

[32] Tien P.-C., Quan M., Kuang S. (2020). Sustained activation of notch signaling maintains tumor-initiating cells in a murine model of liposarcoma. Cancer Lett..

[33] Chen Z., Wang S., Li H.-L., Luo H., Wu X., Lu J., Wang H.-W., Chen Y., Chen D., Wu W.-T. (2022). Fosl1 promotes proneural-to-mesenchymal transition of glioblastoma stem cells via ubc9/cyld/nf-κb axis. Mol. Ther..

[34] Ma D.-Q., Zhang Y.-H., Ding D.-P., Li J., Chen L.-L., Tian Y.-Y., Ao K.-J. (2018). Effect of bmi-1-mediated nf-κb signaling pathway on the stem-like properties of cd133+ human liver cancer cells. Cancer Biomark..

[35] Gonzalez-Torres C., Gaytan-Cervantes J., Vazquez-Santillan K., Mandujano-Tinoco E.A., Ceballos-Cancino G., Garcia-Venzor A., Zampedri C., Sanchez-Maldonado P., Mojica-Espinosa R., Jimenez-Hernandez L.E. (2017). Nf-κb participates in the stem cell phenotype of ovarian cancer cells. Arch. Med. Res..

[36] Kong L., Guo S., Liu C., Zhao Y., Feng C., Liu Y., Wang T., Li C. (2016). Overexpression of sdf-1 activates the nf-κb pathway to induce epithelial to mesenchymal transition and cancer stem cell-like phenotypes of breast cancer cells. Int. J. Oncol..

[37] Li J., Condello S., Thomes-Pepin J., Ma X., Xia Y., Hurley T.D., Matei D., Cheng J.-X. (2017). Lipid desaturation is a metabolic marker and therapeutic target of ovarian cancer stem cells. Cell Stem Cell.

[38] Sun M., Yang Z. (2018). Metabolomic studies of live single cancer stem cells using mass spectrometry. Anal. Chem..

[39] Misra S.K., De A., Pan D. (2018). Targeted delivery of stat-3 modulator to breast cancer stem-like cells downregulates a series of stemness genes. Mol. Cancer Ther..

[40] Wang T., Fahrmann J.F., Lee H., Li Y.-J., Tripathi S.C., Yue C., Zhang C., Lifshitz V., Song J., Yuan Y. (2018). Jak/stat3-regulated fatty acid β-oxidation is critical for breast cancer stem cell self-renewal and chemoresistance. Cell Metab..

[41] Liu S., Zhang C., Wang B., Zhang H., Qin G., Li C., Cao L., Gao Q., Ping Y., Zhang K. (2021). Regulatory t cells promote glioma cell stemness through tgf-β–nf-κb–il6–stat3 signaling. Cancer Immunol. Immunother..

[42] Choi K.M., Kim J.J., Yoo J., Kim K.S., Gu Y., Eom J., Jeong H., Kim K., Nam K.T., Park Y.S. (2022). The interferon-inducible protein viperin controls cancer metabolic reprogramming to enhance cancer progression. J. Clin. Investig..

[43] Wen H., Qian M., He J., Li M., Yu Q., Leng Z. (2020). Inhibiting of self-renewal, migration and invasion of ovarian cancer stem cells by blocking tgf-β pathway. PLoS ONE.

[44] You X., Zhou Z., Chen W., Wei X., Zhou H., Luo W. (2020). Microrna-495 confers inhibitory effects on cancer stem cells in oral squamous cell carcinoma through the hoxc6-mediated tgf-β signaling pathway. Stem Cell Res. Ther..

[45] Chen Z., Chen Y., Li Y., Lian W., Zheng K., Zhang Y., Zhang Y., Lin C., Liu C., Sun F. (2021). Prrx1 promotes stemness and angiogenesis via activating tgf-β/smad pathway and upregulating proangiogenic factors in glioma. Cell Death Dis..

[46] Hassan G., Du J., Afify S.M., Seno A., Seno M. (2020). Cancer stem cell generation by silenced mapk enhancing pi3k/akt signaling. Med. Hypotheses.

[47] Almozyan S., Colak D., Mansour F., Alaiya A., Al-Harazi O., Qattan A., Al-Mohanna F., Al-Alwan M., Ghebeh H. (2017). Pd-l1 promotes oct4 and nanog expression in breast cancer stem cells by sustaining pi3k/akt pathway activation. Int. J. Cancer.

[48] Wang Y.-H., Chan Y.-T., Hung T.-H., Hung J.-T., Kuo M.-W., Wang S.-H., Huang Y., Lin Y.-J., Chen S.-C., Yu J.-C. (2021). Transmembrane and coiled-coil domain family 3 (tmcc3) regulates breast cancer stem cell and akt activation. Oncogene.

[49] Li H., Wang B., Qi B., Jiang G., Qin M., Yu M. (2022). Connexin32 regulates expansion of liver cancer stem cells via the pi3k/akt signaling pathway. Oncol. Rep..

[50] Wang J.H., Gong C., Guo F.J., Zhou X., Zhang M.S., Qiu H., Chao T.F., Liu Y., Qin L., Xiong H.H. (2020). Knockdown of stip1 inhibits the invasion of cd133-positive cancer stem-like cells of the osteosarcoma mg63 cell line via the pi3k/akt and erk1/2 pathways. Int. J. Mol. Med..

[51] Danhier P., Bański P., Payen V.L., Grasso D., Ippolito L., Sonveaux P., Porporato P.E. (2017). Cancer metabolism in space and time: Beyond the warburg effect. Biochim. Biophys. Acta (BBA)-Bioenerg..

[52] Chae Y.C., Kim J.H. (2018). Cancer stem cell metabolism: Target for cancer therapy. BMB Rep..

[53] Butler L.M., Perone Y., Dehairs J., Lupien L.E., de Laat V., Talebi A., Loda M., Kinlaw W.B., Swinnen J.V. (2020). Lipids and cancer: Emerging roles in pathogenesis, diagnosis and therapeutic intervention. Adv. Drug Deliv. Rev..

[54] Koundouros N., Poulogiannis G. (2020). Reprogramming of fatty acid metabolism in cancer. Br. J. Cancer.

[55] Riester M., Xu Q., Moreira A., Zheng J., Michor F., Downey R. (2018). The warburg effect: Persistence of stem-cell metabolism in cancers as a failure of differentiation. Ann. Oncol..

[56] Liu H., Zhang Z., Song L., Gao J., Liu Y. (2022). Lipid metabolism of cancer stem cells. Oncol. Lett..

[57] Huo W., Du M., Pan X., Zhu X., Li Z. (2015). Prognostic value of aldh1 expression in lung cancer: A meta-analysis. Int. J. Clin. Exp. Med..

[58] Guan X., Dong Y., Fan Z., Zhan Y., Xie X., Xu G., Zhang Y., Guo G., Shi A. (2021). Aldehyde dehydrogenase 1 (aldh1) immunostaining in axillary lymph node metastases is an independent prognostic factor in aldh1-positive breast cancer. J. Int. Med. Res..

[59] Xia J., Li S., Liu S., Zhang L. (2023). Aldehyde dehydrogenase in solid tumors and other diseases: Potential biomarkers and therapeutic targets. MedComm.

[60] Mahmood N.A., Abdulghany Z.S., Al-Sudani I.M. (2018). Expression of aldehyde dehydrogenase (aldh1) and atp binding cassette transporter g2 (abcg2) in iraqi patients with colon cancer and the relation with clinicopathological features. Int. J. Mol. Cell. Med..

[61] Corominas-Faja B., Cuyàs E., Gumuzio J., Bosch-Barrera J., Leis O., Martin Á.G., Menendez J.A. (2014). Chemical inhibition of acetyl-coa carboxylase suppresses self-renewal growth of cancer stem cells. Oncotarget.

[62] Pastò A., Bellio C., Pilotto G., Ciminale V., Silic-Benussi M., Guzzo G., Rasola A., Frasson C., Nardo G., Zulato E. (2014). Cancer stem cells from epithelial ovarian cancer patients privilege oxidative phosphorylation, and resist glucose deprivation. Oncotarget.

[63] Yasumoto Y., Miyazaki H., Vaidyan L.K., Kagawa Y., Ebrahimi M., Yamamoto Y., Ogata M., Katsuyama Y., Sadahiro H., Suzuki M. (2016). Inhibition of fatty acid synthase decreases expression of stemness markers in glioma stem cells. PLoS ONE.

[64] Liao T., Kaufmann A.M., Qian X., Sangvatanakul V., Chen C., Kube T., Zhang G., Albers A.E. (2013). Susceptibility to cytotoxic t cell lysis of cancer stem cells derived from cervical and head and neck tumor cell lines. J. Cancer Res. Clin. Oncol..

[65] Kridel S.J., Axelrod F., Rozenkrantz N., Smith J.W. (2004). Orlistat is a novel inhibitor of fatty acid synthase with antitumor activity. Cancer Res..

[66] Chuang H.-Y., Chang Y.-F., Hwang J.-J. (2011). Antitumor effect of orlistat, a fatty acid synthase inhibitor, is via activation of caspase-3 on human colorectal carcinoma-bearing animal. Biomed. Pharmacother..

[67] Zaytseva Y.Y., Rychahou P.G., Le A.-T., Scott T.L., Flight R.M., Kim J.T., Harris J., Liu J., Wang C., Morris A.J. (2018). Preclinical evaluation of novel fatty acid synthase inhibitors in primary colorectal cancer cells and a patient-derived xenograft model of colorectal cancer. Oncotarget.

[68] Cheah F.K., Leong K.H., Thomas N.F., Chin H.K., Ariffin A., Awang K. (2018). Resveratrol analogue,(e)-n-(2-(4-methoxystyryl) phenyl) furan-2-carboxamide induces g 2/m cell cycle arrest through the activation of p53–p21 cip1/waf1 in human colorectal hct116 cells. Apoptosis.

[69] Evans W.J., Morley J.E., Argilés J., Bales C., Baracos V., Guttridge D., Jatoi A., Kalantar-Zadeh K., Lochs H., Mantovani G. (2008). Cachexia: A new definition. Clin. Nutr..

[70] Dahlman I., Mejhert N., Linder K., Agustsson T., Mutch D., Kulyte A., Isaksson B., Permert J., Petrovic N., Nedergaard J. (2010). Adipose tissue pathways involved in weight loss of cancer cachexia. Br. J. Cancer.

[71] Gercel-Taylor C., Doering D.L., Kraemer F.B., Taylor D.D. (1996). Aberrations in normal systemic lipid metabolism in ovarian cancer patients. Gynecol. Oncol..

[72] Menard J.A., Christianson H.C., Kucharzewska P., Bourseau-Guilmain E., Svensson K.J., Lindqvist E., Chandran V.I., Kjellén L., Welinder C., Bengzon J. (2016). Metastasis stimulation by hypoxia and acidosis-induced extracellular lipid uptake is mediated by proteoglycan-dependent endocytosis. Cancer Res..

[73] Tirinato L., Liberale C., Di Franco S., Candeloro P., Benfante A., La Rocca R., Potze L., Marotta R., Ruffilli R., Rajamanickam V.P. (2015). Lipid droplets: A new player in colorectal cancer stem cells unveiled by spectroscopic imaging. Stem Cells.

[74] Lue H.-w., Podolak J., Kolahi K., Cheng L., Rao S., Garg D., Xue C.-H., Rantala J.K., Tyner J.W., Thornburg K.L. (2017). Metabolic reprogramming ensures cancer cell survival despite oncogenic signaling blockade. Genes Dev..

[75] Du W., Zhang L., Brett-Morris A., Aguila B., Kerner J., Hoppel C.L., Puchowicz M., Serra D., Herrero L., Rini B.I. (2017). Hif drives lipid deposition and cancer in ccrcc via repression of fatty acid metabolism. Nat. Commun..

[76] Wang Y.-n., Zeng Z.-l., Lu J., Wang Y., Liu Z.-x., He M.-m., Zhao Q., Wang Z.-x., Li T., Lu Y.-x. (2018). Cpt1a-mediated fatty acid oxidation promotes colorectal cancer cell metastasis by inhibiting anoikis. Oncogene.

[77] Ma L., Chen C., Zhao C., Li T., Ma L., Jiang J., Duan Z., Si Q., Chuang T.-H., Xiang R. (2024). Targeting carnitine palmitoyl transferase 1a (cpt1a) induces ferroptosis and synergizes with immunotherapy in lung cancer. Signal Transduct. Target. Ther..

[78] Yan F., Zhao H., Zeng Y. (2018). Lipidomics: A promising cancer biomarker. Clin. Transl. Med..

[79] Nakamura M.T., Nara T.Y. (2004). Structure, function, and dietary regulation of δ6, δ5, and δ9 desaturases. Annu. Rev. Nutr..

[80] Castro L.F.C., Wilson J.M., Gonçalves O., Galante-Oliveira S., Rocha E., Cunha I. (2011). The evolutionary history of the stearoyl-coa desaturase gene family in vertebrates. BMC Evol. Biol..

[81] Carracedo A., Cantley L.C., Pandolfi P.P. (2013). Cancer metabolism: Fatty acid oxidation in the limelight. Nat. Rev. Cancer.

[82] Liu L., Zhao X., Zhao L., Li J., Yang H., Zhu Z., Liu J., Huang G. (2016). Arginine methylation of srebp1a via prmt5 promotes de novo lipogenesis and tumor growth. Cancer Res..

[83] Schmidt D.R., Patel R., Kirsch D.G., Lewis C.A., Vander Heiden M.G., Locasale J.W. (2021). Metabolomics in cancer research and emerging applications in clinical oncology. CA A Cancer J. Clin..

[84] Yadav S., Virk R., Chung C.H., Eduardo M.B., VanDerway D., Chen D., Burdett K., Gao H., Zeng Z., Ranjan M. (2022). Lipid exposure activates gene expression changes associated with estrogen receptor negative breast cancer. npj Breast Cancer.

[85] Corbet C., Feron O. (2017). Emerging roles of lipid metabolism in cancer progression. Curr. Opin. Clin. Nutr. Metab. Care.

[86] Kusama T., Mukai M., Iwasaki T., Tatsuta M., Matsumoto Y., Akedo H., Inoue M., Nakamura H. (2002). 3-hydroxy-3-methylglutaryl-coenzyme a reductase inhibitors reduce human pancreatic cancer cell invasion and metastasis. Gastroenterology.

[87] Gupta G.P., Nguyen D.X., Chiang A.C., Bos P.D., Kim J.Y., Nadal C., Gomis R.R., Manova-Todorova K., Massagué J. (2007). Mediators of vascular remodelling co-opted for sequential steps in lung metastasis. Nature.

[88] Ramírez de Molina A., Gutiérrez R., Ramos M.A., Silva J.M., Silva J., Bonilla F., Sánchez J.J., Lacal J.C. (2002). Increased choline kinase activity in human breast carcinomas: Clinical evidence for a potential novel antitumor strategy. Oncogene.

[89] Iorio E., Ricci A., Bagnoli M., Pisanu M.E., Castellano G., Di Vito M., Venturini E., Glunde K., Bhujwalla Z.M., Mezzanzanica D. (2010). Activation of phosphatidylcholine cycle enzymes in human epithelial ovarian cancer cells. Cancer Res..

[90] Sung G.-J., Choi H.-K., Kwak S., Song J.-H., Ko H., Yoon H.-G., Kang H.-B., Choi K.-C. (2016). Targeting cpt1a enhances metabolic therapy in human melanoma cells with the braf v600e mutation. Int. J. Biochem. Cell Biol..

[91] Cheng X., Geng F., Guo D. (2020). Dgat1 protects tumor from lipotoxicity, emerging as a promising metabolic target for cancer therapy. Mol. Cell. Oncol..

[92] Alphey M.S., Yu W., Byres E., Li D., Hunter W.N. (2005). Structure and reactivity of human mitochondrial 2, 4-dienoyl-coa reductase: Enzyme-ligand interactions in a distinctive short-chain reductase active site. J. Biol. Chem..

[93] Sánchez-Martínez R., Cruz-Gil S., de Cedrón M.G., Álvarez-Fernández M., Vargas T., Molina S., García B., Herranz J., Moreno-Rubio J., Reglero G. (2015). A link between lipid metabolism and epithelial-mesenchymal transition provides a target for colon cancer therapy. Oncotarget.

[94] Yang W.S., Kim K.J., Gaschler M.M., Patel M., Shchepinov M.S., Stockwell B.R. (2016). Peroxidation of polyunsaturated fatty acids by lipoxygenases drives ferroptosis. Proc. Natl. Acad. Sci. USA.

[95] Yan C., Zhao T., Du H. (2015). Lysosomal acid lipase in cancer. Oncoscience.

[96] Shao N., Qiu H., Liu J., Xiao D., Zhao J., Chen C., Wan J., Guo M., Liang G., Zhao X. (2024). Targeting lipid metabolism of macrophages: A new strategy for tumor therapy. J. Adv. Res..

[97] Min J.-Y., Kim D.-H. (2023). Stearoyl-coa desaturase 1 as a therapeutic biomarker: Focusing on cancer stem cells. Int. J. Mol. Sci..

[98] Li M., Tang Y., Wang D., Zhai X., Shen H., Zhong C., Yao M., Jin A., Zhou Z., Zhou S. (2022). Sphingosine-1-phosphate transporter spinster homolog 2 is essential for iron-regulated metastasis of hepatocellular carcinoma. Mol. Ther..

[99] Su F., Koeberle A. (2023). Regulation and targeting of srebp-1 in hepatocellular carcinoma. Cancer Metastasis Rev..

[100] Sharon C., Baranwal S., Patel N.J., Rodriguez-Agudo D., Pandak W.M., Majumdar A.P., Krystal G., Patel B.B. (2015). Inhibition of insulin-like growth factor receptor/akt/mammalian target of rapamycin axis targets colorectal cancer stem cells by attenuating mevalonate-isoprenoid pathway in vitro and in vivo. Oncotarget.

[101] Goyette S., Liang Y., Mafuvadze B., Cook M.T., Munir M., Hyder S.M. (2017). Natural and synthetic progestins enrich cancer stem cell-like cells in hormone-responsive human breast cancer cell populations in vitro. Breast Cancer Targets Ther..

[102] Hu C., Xin Z., Sun X., Hu Y., Zhang C., Yan R., Wang Y., Lu M., Huang J., Du X. (2023). Activation of acly by sec63 deploys metabolic reprogramming to facilitate hepatocellular carcinoma metastasis upon endoplasmic reticulum stress. J. Exp. Clin. Cancer Res..

[103] He W., Liang B., Wang C., Li S., Zhao Y., Huang Q., Liu Z., Yao Z., Wu Q., Liao W. (2019). Msc-regulated lncrna macc1-as1 promotes stemness and chemoresistance through fatty acid oxidation in gastric cancer. Oncogene.

[104] Mascaraque M., Courtois S., Royo-García A., Barneda D., Stoian A.M., Villaoslada I., Espiau-Romera P., Bokil A., Cano-Galiano A., Jagust P. (2024). Fatty acid oxidation is critical for the tumorigenic potential and chemoresistance of pancreatic cancer stem cells. J. Transl. Med..

[105] Park J.H., Vithayathil S., Kumar S., Sung P.-L., Dobrolecki L.E., Putluri V., Bhat V.B., Bhowmik S.K., Gupta V., Arora K. (2016). Fatty acid oxidation-driven src links mitochondrial energy reprogramming and oncogenic properties in triple-negative breast cancer. Cell Rep..

[106] Lee E.A., Angka L., Rota S.-G., Hanlon T., Mitchell A., Hurren R., Wang X.M., Gronda M., Boyaci E., Bojko B. (2015). Targeting mitochondria with avocatin b induces selective leukemia cell death. Cancer Res..

[107] Dixon S.J., Lemberg K.M., Lamprecht M.R., Skouta R., Zaitsev E.M., Gleason C.E., Patel D.N., Bauer A.J., Cantley A.M., Yang W.S. (2012). Ferroptosis: An iron-dependent form of nonapoptotic cell death. Cell.

[108] Kagan V.E., Mao G., Qu F., Angeli J.P.F., Doll S., Croix C.S., Dar H.H., Liu B., Tyurin V.A., Ritov V.B. (2017). Oxidized arachidonic and adrenic pes navigate cells to ferroptosis. Nat. Chem. Biol..

[109] Zou Y., Henry W.S., Ricq E.L., Graham E.T., Phadnis V.V., Maretich P., Paradkar S., Boehnke N., Deik A.A., Reinhardt F. (2020). Plasticity of ether lipids promotes ferroptosis susceptibility and evasion. Nature.

[110] Phadnis V.V., Snider J., Varadharajan V., Ramachandiran I., Deik A.A., Lai Z.W., Kunchok T., Eaton E.N., Sebastiany C., Lyakisheva A. (2023). Mmd collaborates with acsl4 and mboat7 to promote polyunsaturated phosphatidylinositol remodeling and susceptibility to ferroptosis. Cell Rep..

[111] Possik E., Jalali Z., Nouët Y., Yan M., Gingras M.-C., Schmeisser K., Panaite L., Dupuy F., Kharitidi D., Chotard L. (2014). Folliculin regulates ampk-dependent autophagy and metabolic stress survival. PLoS Genet..

[112] She C., Zhu L.-Q., Zhen Y.-F., Wang X.-D., Dong Q.-R. (2014). Activation of ampk protects against hydrogen peroxide-induced osteoblast apoptosis through autophagy induction and nadph maintenance: New implications for osteonecrosis treatment?. Cell. Signal..

[113] Porstmann T., Santos C.R., Griffiths B., Cully M., Wu M., Leevers S., Griffiths J.R., Chung Y.-L., Schulze A. (2008). Srebp activity is regulated by mtorc1 and contributes to akt-dependent cell growth. Cell Metab..

[114] Keerthana C.K., Rayginia T.P., Shifana S.C., Anto N.P., Kalimuthu K., Isakov N., Anto R.J. (2023). The role of ampk in cancer metabolism and its impact on the immunomodulation of the tumor microenvironment. Front. Immunol..

[115] Kovale L., Singh M.K., Kim J., Ha J. (2024). Role of autophagy and ampk in cancer stem cells: Therapeutic opportunities and obstacles in cancer. Int. J. Mol. Sci..

[116] Reya T., Morrison S.J., Clarke M.F., Weissman I.L. (2001). Stem cells, cancer, and cancer stem cells. Nature.

[117] Olzmann J.A., Carvalho P. (2019). Dynamics and functions of lipid droplets. Nat. Rev. Mol. Cell Biol..

[118] Hoxhaj G., Manning B.D. (2020). The pi3k–akt network at the interface of oncogenic signalling and cancer metabolism. Nat. Rev. Cancer.

[119] Dubrovska A., Kim S., Salamone R.J., Walker J.R., Maira S.-M., García-Echeverría C., Schultz P.G., Reddy V.A. (2009). The role of pten/akt/pi3k signaling in the maintenance and viability of prostate cancer stem-like cell populations. Proc. Natl. Acad. Sci. USA.

[120] Song N.-J., Yun U.J., Yang S., Wu C., Seo C.-R., Gwon A.-R., Baik S.-H., Choi Y., Choi B.Y., Bahn G. (2016). Notch1 deficiency decreases hepatic lipid accumulation by induction of fatty acid oxidation. Sci. Rep..

[121] Yu Y., Kim H., Choi S., Yu J., Lee J.Y., Lee H., Yoon S., Kim W.-Y. (2021). Targeting a lipid desaturation enzyme, scd1, selectively eliminates colon cancer stem cells through the suppression of wnt and notch signaling. Cells.

[122] Kalucka J., Bierhansl L., Conchinha N.V., Missiaen R., Elia I., Brüning U., Scheinok S., Treps L., Cantelmo A.R., Dubois C. (2018). Quiescent endothelial cells upregulate fatty acid β-oxidation for vasculoprotection via redox homeostasis. Cell Metab..

[123] Mi W., Lin Q., Childress C., Sudol M., Robishaw J., Berlot C., Shabahang M., Yang W. (2015). Geranylgeranylation signals to the hippo pathway for breast cancer cell proliferation and migration. Oncogene.

[124] Lin Q., Yang W. (2016). The hippo-yap/taz pathway mediates geranylgeranylation signaling in breast cancer progression. Mol. Cell. Oncol..

[125] Cordenonsi M., Zanconato F., Azzolin L., Forcato M., Rosato A., Frasson C., Inui M., Montagner M., Parenti A.R., Poletti A. (2011). The hippo transducer taz confers cancer stem cell-related traits on breast cancer cells. Cell.

[126] Huo X., Zhang Q., Liu A.M., Tang C., Gong Y., Bian J., Luk J.M., Xu Z., Chen J. (2013). Overexpression of yes-associated protein confers doxorubicin resistance in hepatocellullar carcinoma. Oncol. Rep..

[127] Zanconato F., Cordenonsi M., Piccolo S. (2016). Yap/taz at the roots of cancer. Cancer Cell.

[128] Noto A., De Vitis C., Pisanu M., Roscilli G., Ricci G., Catizone A., Sorrentino G., Chianese G., Taglialatela-Scafati O., Trisciuoglio D. (2017). Stearoyl-coa-desaturase 1 regulates lung cancer stemness via stabilization and nuclear localization of yap/taz. Oncogene.

[129] Noto A., Raffa S., De Vitis C., Roscilli G., Malpicci D., Coluccia P., Di Napoli A., Ricci A., Giovagnoli M.R., Aurisicchio L. (2013). Stearoyl-coa desaturase-1 is a key factor for lung cancer-initiating cells. Cell Death Dis..

[130] Cook A.M., Li L., Ho Y., Lin A., Li L., Stein A., Forman S., Perrotti D., Jove R., Bhatia R. (2014). Role of altered growth factor receptor-mediated jak2 signaling in growth and maintenance of human acute myeloid leukemia stem cells. Blood J. Am. Soc. Hematol..

[131] Fendler A., Bauer D., Busch J., Jung K., Wulf-Goldenberg A., Kunz S., Song K., Myszczyszyn A., Elezkurtaj S., Erguen B. (2020). Inhibiting wnt and notch in renal cancer stem cells and the implications for human patients. Nat. Commun..

[132] Fu T., Coulter S., Yoshihara E., Oh T.G., Fang S., Cayabyab F., Zhu Q., Zhang T., Leblanc M., Liu S. (2019). Fxr regulates intestinal cancer stem cell proliferation. Cell.

[133] Vergara D., Stanca E., Guerra F., Priore P., Gaballo A., Franck J., Simeone P., Trerotola M., De Domenico S., Fournier I. (2017). Β-catenin knockdown affects mitochondrial biogenesis and lipid metabolism in breast cancer cells. Front. Physiol..

[134] Bersuker K., Hendricks J.M., Li Z., Magtanong L., Ford B., Tang P.H., Roberts M.A., Tong B., Maimone T.J., Zoncu R. (2019). The coq oxidoreductase fsp1 acts parallel to gpx4 to inhibit ferroptosis. Nature.

[135] Doll S., Freitas F.P., Shah R., Aldrovandi M., da Silva M.C., Ingold I., Goya Grocin A., Xavier da Silva T.N., Panzilius E., Scheel C.H. (2019). Fsp1 is a glutathione-independent ferroptosis suppressor. Nature.

[136] Mao C., Liu X., Zhang Y., Lei G., Yan Y., Lee H., Koppula P., Wu S., Zhuang L., Fang B. (2021). Dhodh-mediated ferroptosis defence is a targetable vulnerability in cancer. Nature.

[137] Kraft V.A., Bezjian C.T., Pfeiffer S., Ringelstetter L., Müller C., Zandkarimi F., Merl-Pham J., Bao X., Anastasov N., Kössl J. (2019). Gtp cyclohydrolase 1/tetrahydrobiopterin counteract ferroptosis through lipid remodeling. ACS Cent. Sci..

[138] Dodson M., Castro-Portuguez R., Zhang D.D. (2019). Nrf2 plays a critical role in mitigating lipid peroxidation and ferroptosis. Redox Biol..

[139] Guen V.J., Chavarria T.E., Kröger C., Ye X., Weinberg R.A., Lees J.A. (2017). Emt programs promote basal mammary stem cell and tumor-initiating cell stemness by inducing primary ciliogenesis and hedgehog signaling. Proc. Natl. Acad. Sci. USA.

[140] Li N., Singh S., Cherukuri P., Li H., Yuan Z., Ellisen L.W., Wang B., Robbins D., DiRenzo J. (2008). Reciprocal intraepithelial interactions between tp63 and hedgehog signaling regulate quiescence and activation of progenitor elaboration by mammary stem cells. Stem Cells.

[141] Chen J.K., Taipale J., Cooper M.K., Beachy P.A. (2002). Inhibition of hedgehog signaling by direct binding of cyclopamine to smoothened. Genes Dev..

[142] Mazumdar T., DeVecchio J., Agyeman A., Shi T., Houghton J.A. (2011). Blocking hedgehog survival signaling at the level of the gli genes induces DNA damage and extensive cell death in human colon carcinoma cells. Cancer Res..

[143] Desch P., Asslaber D., Kern D., Schnidar H., Mangelberger D., Alinger B., Stoecher M., Hofbauer S., Neureiter D., Tinhofer I. (2010). Inhibition of gli, but not smoothened, induces apoptosis in chronic lymphocytic leukemia cells. Oncogene.

[144] Long B., Wang L.-X., Zheng F.-M., Lai S.-P., Xu D.-R., Hu Y., Lin D.-J., Zhang X.-Z., Dong L., Long Z.-J. (2016). Targeting gli1 suppresses cell growth and enhances chemosensitivity in cd34+ enriched acute myeloid leukemia progenitor cells. Cell. Physiol. Biochem..

[145] Choi A.M., Ryter S.W., Levine B. (2013). Autophagy in human health and disease. N. Engl. J. Med..

[146] Mizushima N., Komatsu M. (2011). Autophagy: Renovation of cells and tissues. Cell.

[147] García-Prat L., Martínez-Vicente M., Perdiguero E., Ortet L., Rodríguez-Ubreva J., Rebollo E., Ruiz-Bonilla V., Gutarra S., Ballestar E., Serrano A.L. (2016). Autophagy maintains stemness by preventing senescence. Nature.

[148] Huang F., Wang B.-R., Wang Y.-G. (2018). Role of autophagy in tumorigenesis, metastasis, targeted therapy and drug resistance of hepatocellular carcinoma. World J. Gastroenterol..

[149] Sharif T., Martell E., Dai C., Kennedy B.E., Murphy P., Clements D.R., Kim Y., Lee P.W., Gujar S.A. (2017). Autophagic homeostasis is required for the pluripotency of cancer stem cells. Autophagy.

[150] Yoshida G.J. (2017). Therapeutic strategies of drug repositioning targeting autophagy to induce cancer cell death: From pathophysiology to treatment. J. Hematol. Oncol..

[151] Viale A., Pettazzoni P., Lyssiotis C.A., Ying H., Sánchez N., Marchesini M., Carugo A., Green T., Seth S., Giuliani V. (2014). Oncogene ablation-resistant pancreatic cancer cells depend on mitochondrial function. Nature.

[152] Sunayama J., Sato A., Matsuda K.-I., Tachibana K., Watanabe E., Seino S., Suzuki K., Narita Y., Shibui S., Sakurada K. (2011). Foxo3a functions as a key integrator of cellular signals that control glioblastoma stem-like cell differentiation and tumorigenicity. Stem Cells.

[153] Prabhu V.V., Allen J.E., Dicker D.T., El-Deiry W.S. (2015). Small-molecule onc201/tic10 targets chemotherapy-resistant colorectal cancer stem–like cells in an akt/foxo3a/trail–dependent manner. Cancer Res..

[154] Ning Y., Luo C., Ren K., Quan M., Cao J. (2021). Foxo3a-mediated suppression of the self-renewal capacity of sphere-forming cells derived from the ovarian cancer skov3 cell line by 7-difluoromethoxyl-5, 4′-di-n-octyl genistein. Mol. Med. Rep..

[155] Peng Q., Qin J., Zhang Y., Cheng X., Wang X., Lu W., Xie X., Zhang S. (2017). Autophagy maintains the stemness of ovarian cancer stem cells by foxa2. J. Exp. Clin. Cancer Res..

[156] Cufí S., Vazquez-Martin A., Oliveras-Ferraros C., Martin-Castillo B., Vellon L., Menendez J.A. (2011). Autophagy positively regulates the cd44+ cd24-/low breast cancer stem-like phenotype. Cell Cycle.

[157] Ito K., Turcotte R., Cui J., Zimmerman S.E., Pinho S., Mizoguchi T., Arai F., Runnels J.M., Alt C., Teruya-Feldstein J. (2016). Self-renewal of a purified tie2+ hematopoietic stem cell population relies on mitochondrial clearance. Science.

[158] Sinha R.A., Singh B.K., Zhou J., Wu Y., Farah B.L., Ohba K., Lesmana R., Gooding J., Bay B.-H., Yen P.M. (2015). Thyroid hormone induction of mitochondrial activity is coupled to mitophagy via ros-ampk-ulk1 signaling. Autophagy.

[159] Nguyen H., Yang J., Kung H., Shi X., Tilki D., Lara P., DeVere White R., Gao A., Evans C. (2014). Targeting autophagy overcomes enzalutamide resistance in castration-resistant prostate cancer cells and improves therapeutic response in a xenograft model. Oncogene.

[160] Sun R., Shen S., Zhang Y.-J., Xu C.-F., Cao Z.-T., Wen L.-P., Wang J. (2016). Nanoparticle-facilitated autophagy inhibition promotes the efficacy of chemotherapeutics against breast cancer stem cells. Biomaterials.

[161] Lu L., Shen X., Tao B., Lin C., Li K., Luo Z., Cai K. (2019). The nanoparticle-facilitated autophagy inhibition of cancer stem cells for improved chemotherapeutic effects on glioblastomas. J. Mater. Chem. B.

[162] Gupta A., Roy S., Lazar A.J., Wang W.-L., McAuliffe J.C., Reynoso D., McMahon J., Taguchi T., Floris G., Debiec-Rychter M. (2010). Autophagy inhibition and antimalarials promote cell death in gastrointestinal stromal tumor (gist). Proc. Natl. Acad. Sci. USA.

[163] Bai Y., Meng L., Han L., Jia Y., Zhao Y., Gao H., Kang R., Wang X., Tang D., Dai E. (2019). Lipid storage and lipophagy regulates ferroptosis. Biochem. Biophys. Res. Commun..

[164] Singh R., Kaushik S., Wang Y., Xiang Y., Novak I., Komatsu M., Tanaka K., Cuervo A.M., Czaja M.J. (2009). Autophagy regulates lipid metabolism. Nature.

[165] Farah B.L., Landau D.J., Sinha R.A., Brooks E.D., Wu Y., Fung S.Y.S., Tanaka T., Hirayama M., Bay B.-H., Koeberl D.D. (2016). Induction of autophagy improves hepatic lipid metabolism in glucose-6-phosphatase deficiency. J. Hepatol..

[166] Zhao T., Du H., Ding X., Walls K., Yan C. (2015). Activation of mtor pathway in myeloid-derived suppressor cells stimulates cancer cell proliferation and metastasis in lal−/− mice. Oncogene.

[167] Bort Bueno A.C., Sánchez Gómez B., Miguel García I.d., Mateos Gómez P.A., Díaz-Laviada Marturet I.C. (2020). Dysregulated lipid metabolism in hepatocellular carcinoma cancer stem cells. Mol. Biol. Rep..

[168] DePeralta D.K., Wei L., Harriman G., Greenwood J., Bhat S., Westlin W., Harwood H.J., Kapeller R., Tanabe K.K., Fuchs B.C. (2014). Liver selective acetyl-coa carboxylase inhibition by nd-654 decreases hepatocellular carcinoma development in cirrhotic rats. Cancer Res..

[169] Svensson R., Harriman G., Greenwood J., Bhat S., Harwood H.J., Kapeller R., Shaw R. (2014). Acetyl-coa carboxylase inhibition by nd646 reduces fatty acid synthesis and inhibits cell proliferation in human non-small cell lung cancer cells. Cancer Res..

[170] Liang K. (2023). Mitochondrial cpt1a: Insights into structure, function, and basis for drug development. Front. Pharmacol..

[171] Yu H., Lee H., Herrmann A., Buettner R., Jove R. (2014). Revisiting stat3 signalling in cancer: New and unexpected biological functions. Nat. Rev. Cancer.

[172] Liu C., Qiang J., Deng Q., Xia J., Deng L., Zhou L., Wang D., He X., Liu Y., Zhao B. (2021). Aldh1a1 activity in tumor-initiating cells remodels myeloid-derived suppressor cells to promote breast cancer progression. Cancer Res..

[173] Formenti S.C., Hawtin R.E., Dixit N., Evensen E., Lee P., Goldberg J.D., Li X., Vanpouille-Box C., Schaue D., McBride W.H. (2019). Baseline t cell dysfunction by single cell network profiling in metastatic breast cancer patients. J. Immunother. Cancer.

[174] Cole A.J., Fayomi A.P., Anyaeche V.I., Bai S., Buckanovich R.J. (2020). An evolving paradigm of cancer stem cell hierarchies: Therapeutic implications. Theranostics.

[175] Cortes J.E., Heidel F.H., Hellmann A., Fiedler W., Smith B.D., Robak T., Montesinos P., Pollyea D.A., DesJardins P., Ottmann O. (2019). Randomized comparison of low dose cytarabine with or without glasdegib in patients with newly diagnosed acute myeloid leukemia or high-risk myelodysplastic syndrome. Leukemia.

[176] Danial C., Sarin K.Y., Oro A.E., Chang A.L.S. (2016). An investigator-initiated open-label trial of sonidegib in advanced basal cell carcinoma patients resistant to vismodegib. Clin. Cancer Res..

[177] Chushi L., Wei W., Kangkang X., Yongzeng F., Ning X., Xiaolei C. (2016). Hmgcr is up-regulated in gastric cancer and promotes the growth and migration of the cancer cells. Gene.

[178] Zaidi N., Royaux I., Swinnen J.V., Smans K. (2012). Atp citrate lyase knockdown induces growth arrest and apoptosis through different cell-and environment-dependent mechanisms. Mol. Cancer Ther..

[179] Wei X., Shi J., Lin Q., Ma X., Pang Y., Mao H., Li R., Lu W., Wang Y., Liu P. (2021). Targeting acly attenuates tumor growth and acquired cisplatin resistance in ovarian cancer by inhibiting the pi3k–akt pathway and activating the ampk–ros pathway. Front. Oncol..

[180] Schug Z.T., Peck B., Jones D.T., Zhang Q., Grosskurth S., Alam I.S., Goodwin L.M., Smethurst E., Mason S., Blyth K. (2015). Acetyl-coa synthetase 2 promotes acetate utilization and maintains cancer cell growth under metabolic stress. Cancer Cell.

[181] Hess D., Chisholm J.W., Igal R.A. (2010). Inhibition of stearoylcoa desaturase activity blocks cell cycle progression and induces programmed cell death in lung cancer cells. PLoS ONE.

[182] Oballa R.M., Belair L., Black W.C., Bleasby K., Chan C.C., Desroches C., Du X., Gordon R., Guay J., Guiral S. (2011). Development of a liver-targeted stearoyl-coa desaturase (scd) inhibitor (mk-8245) to establish a therapeutic window for the treatment of diabetes and dyslipidemia. J. Med. Chem..

[183] Ran H., Zhu Y., Deng R., Zhang Q., Liu X., Feng M., Zhong J., Lin S., Tong X., Su Q. (2018). Stearoyl-coa desaturase-1 promotes colorectal cancer metastasis in response to glucose by suppressing pten. J. Exp. Clin. Cancer Res..

[184] Potze L., Di Franco S., H Kessler J., Stassi G., Paul Medema J. (2016). Betulinic acid kills colon cancer stem cells. Curr. Stem Cell Res. Ther..

[185] El-Sahli S., Xie Y., Wang L., Liu S. (2019). Wnt signaling in cancer metabolism and immunity. Cancers.

[186] De Vitis C., Tabbì E., Mancini R., Raffa S., Torrisi M.R., Pisanu M.E., Fulciniti F., Ascierto P.A., Ciliberto G., Maugeri-Saccà M. (2018). Inhibition of stearoyl-coa desaturase 1 reverts braf and mek inhibition-induced selection of cancer stem cells in braf-mutated melanoma. J. Exp. Clin. Cancer Res..

[187] Choi S., Yoo Y.J., Kim H., Lee H., Chung H., Nam M.-H., Moon J.-Y., Lee H.S., Yoon S., Kim W.-Y. (2019). Clinical and biochemical relevance of monounsaturated fatty acid metabolism targeting strategy for cancer stem cell elimination in colon cancer. Biochem. Biophys. Res. Commun..

[188] Kloosterman D.J., Erbani J., Boon M., Farber M., Handgraaf S.M., Ando-Kuri M., Sánchez-López E., Fontein B., Mertz M., Nieuwland M. (2024). Macrophage-mediated myelin recycling fuels brain cancer malignancy. Cell.

[189] Sevenich L. (2024). Lipid Recycling by Macrophage Cells Drives the Rrowth of Brain Cancer.

[190] Smith A.G., Macleod K.F. (2019). Autophagy, cancer stem cells and drug resistance. J. Pathol..

[191] Martin K.R., Celano S.L., Solitro A.R., Gunaydin H., Scott M., O’Hagan R.C., Shumway S.D., Fuller P., MacKeigan J.P. (2018). A potent and selective ulk1 inhibitor suppresses autophagy and sensitizes cancer cells to nutrient stress. iScience.

[192] Egan D.F., Chun M.G., Vamos M., Zou H., Rong J., Miller C.J., Lou H.J., Raveendra-Panickar D., Yang C.-C., Sheffler D.J. (2015). Small molecule inhibition of the autophagy kinase ulk1 and identification of ulk1 substrates. Mol. Cell.

[193] Petherick K.J., Conway O.J., Mpamhanga C., Osborne S.A., Kamal A., Saxty B., Ganley I.G. (2015). Pharmacological inhibition of ulk1 kinase blocks mammalian target of rapamycin (mtor)-dependent autophagy. J. Biol. Chem..

[194] Chen J.-L., Wu X., Yin D., Jia X.-H., Chen X., Gu Z.-Y., Zhu X.-M. (2023). Autophagy inhibitors for cancer therapy: Small molecules and nanomedicines. Pharmacol. Ther..

[195] Rebecca V.W., Nicastri M.C., Fennelly C., Chude C.I., Barber-Rotenberg J.S., Ronghe A., McAfee Q., McLaughlin N.P., Zhang G., Goldman A.R. (2019). Ppt1 promotes tumor growth and is the molecular target of chloroquine derivatives in cancer. Cancer Discov..

[196] Xu J., Su Z., Cheng X., Hu S., Wang W., Zou T., Zhou X., Song Z., Xia Y., Gao Y. (2022). High ppt1 expression predicts poor clinical outcome and ppt1 inhibitor dc661 enhances sorafenib sensitivity in hepatocellular carcinoma. Cancer Cell Int..

[197] Gao Y., Liu Y., Hong L., Yang Z., Cai X., Chen X., Fu Y., Lin Y., Wen W., Li S. (2016). Golgi-associated lc3 lipidation requires v-atpase in noncanonical autophagy. Cell Death Dis..

[198] Bellodi C., Lidonnici M.R., Hamilton A., Helgason G.V., Soliera A.R., Ronchetti M., Galavotti S., Young K.W., Selmi T., Yacobi R. (2009). Targeting autophagy potentiates tyrosine kinase inhibitor–induced cell death in philadelphia chromosome–positive cells, including primary cml stem cells. J. Clin. Investig..

[199] Zheng K., Li Y., Wang S., Wang X., Liao C., Hu X., Fan L., Kang Q., Zeng Y., Wu X. (2016). Inhibition of autophagosome-lysosome fusion by ginsenoside ro via the esr2-ncf1-ros pathway sensitizes esophageal cancer cells to 5-fluorouracil-induced cell death via the chek1-mediated DNA damage checkpoint. Autophagy.

[200] Kostecka L.G., Mendez S., Li M., Khare P., Zhang C., Le A., Amend S.R., Pienta K.J. (2024). Cancer cells employ lipid droplets to survive toxic stress. Prostate.

[201] Lee S.S.-Y., Li J., Tai J.N., Ratliff T.L., Park K., Cheng J.-X. (2015). Avasimibe encapsulated in human serum albumin blocks cholesterol esterification for selective cancer treatment. ACS Nano.

[202] Theodoropoulos P.C., Gonzales S.S., Winterton S.E., Rodriguez-Navas C., McKnight J.S., Morlock L.K., Hanson J.M., Cross B., Owen A.E., Duan Y. (2016). Discovery of tumor-specific irreversible inhibitors of stearoyl coa desaturase. Nat. Chem. Biol..

[203] Mukherjee A., Chiang C.-Y., Daifotis H.A., Nieman K.M., Fahrmann J.F., Lastra R.R., Romero I.L., Fiehn O., Lengyel E. (2020). Adipocyte-induced fabp4 expression in ovarian cancer cells promotes metastasis and mediates carboplatin resistance. Cancer Res..

[204] Uehara H., Takahashi T., Oha M., Ogawa H., Izumi K. (2014). Exogenous fatty acid binding protein 4 promotes human prostate cancer cell progression. Int. J. Cancer.

[205] Gupta A., Das D., Taneja R. (2024). Targeting dysregulated lipid metabolism in cancer with pharmacological inhibitors. Cancers.

[206] Orita H., Coulter J., Tully E., Abe M., Montgomery E., Alvarez H., Sato K., Hino O., Kajiyama Y., Tsurumaru M. (2010). High levels of fatty acid synthase expression in esophageal cancers represent a potential target for therapy. Cancer Biol. Ther..

[207] Wu H., Han Y., Rodriguez Sillke Y., Deng H., Siddiqui S., Treese C., Schmidt F., Friedrich M., Keye J., Wan J. (2019). Lipid droplet-dependent fatty acid metabolism controls the immune suppressive phenotype of tumor-associated macrophages. EMBO Mol. Med..

[208] Cai Y., Crowther J., Pastor T., Asbagh L.A., Baietti M.F., De Troyer M., Vazquez I., Talebi A., Renzi F., Dehairs J. (2016). Loss of chromosome 8p governs tumor progression and drug response by altering lipid metabolism. Cancer Cell.

[209] Shaykevich A., Chae D., Silverman I., Bassali J., Louloueian N., Siegman A., Bandyopadhyaya G., Goel S., Maitra R. (2024). Impact of carbamazepine on smarca4 (brg1) expression in colorectal cancer: Modulation by kras mutation status. Investig. New Drugs.

[210] Walcher L., Kistenmacher A.-K., Suo H., Kitte R., Dluczek S., Strauß A., Blaudszun A.-R., Yevsa T., Fricke S., Kossatz-Boehlert U. (2020). Cancer stem cells—Origins and biomarkers: Perspectives for targeted personalized therapies. Front. Immunol..

[211] Oh S.J., Noh K.H., Lee Y.-H., Hong S.-O., Song K.-H., Lee H.-J., Kim S., Kim T.M., Jeon J.-H., Seo J.H. (2015). Targeting stemness is an effective strategy to control eml4-alk+ non-small cell lung cancer cells. Oncotarget.

[212] Kazandjian D., Blumenthal G.M., Chen H.-Y., He K., Patel M., Justice R., Keegan P., Pazdur R. (2014). Fda approval summary: Crizotinib for the treatment of metastatic non-small cell lung cancer with anaplastic lymphoma kinase rearrangements. Oncol..

[213] Malvi P., Chaube B., Singh S.V., Mohammad N., Pandey V., Vijayakumar M.V., Radhakrishnan R.M., Vanuopadath M., Nair S.S., Nair B.G. (2016). Weight control interventions improve therapeutic efficacy of dacarbazine in melanoma by reversing obesity-induced drug resistance. Cancer Metab..

[214] Danielpour D. (2024). Advances and challenges in targeting tgf-β isoforms for therapeutic intervention of cancer: A mechanism-based perspective. Pharmaceuticals.

[215] Wu G., Wang Q., Xu Y., Li J., Zhang H., Qi G., Xia Q. (2019). Targeting the transcription factor receptor lxr to treat clear cell renal cell carcinoma: Agonist or inverse agonist?. Cell Death Dis..

[216] Zhou Y., Bollu L.R., Tozzi F., Ye X., Bhattacharya R., Gao G., Dupre E., Xia L., Lu J., Fan F. (2013). Atp citrate lyase mediates resistance of colorectal cancer cells to sn38. Mol. Cancer Ther..

[217] Matsufuji T., Ikeda M., Naito A., Hirouchi M., Kanda S., Izumi M., Harada J., Shinozuka T. (2013). Arylpiperazines as fatty acid transport protein 1 (fatp1) inhibitors with improved potency and pharmacokinetic properties. Bioorganic Med. Chem. Lett..

[218] Hale J.S., Otvos B., Sinyuk M., Alvarado A.G., Hitomi M., Stoltz K., Wu Q., Flavahan W., Levison B., Johansen M.L. (2014). Cancer stem cell-specific scavenger receptor cd36 drives glioblastoma progression. Stem Cells.

[219] Svensson R.U., Parker S.J., Eichner L.J., Kolar M.J., Wallace M., Brun S.N., Lombardo P.S., Van Nostrand J.L., Hutchins A., Vera L. (2016). Inhibition of acetyl-coa carboxylase suppresses fatty acid synthesis and tumor growth of non-small-cell lung cancer in preclinical models. Nat. Med..

[220] Schcolnik-Cabrera A., Chávez-Blanco A., Domínguez-Gómez G., Taja-Chayeb L., Morales-Barcenas R., Trejo-Becerril C., Perez-Cardenas E., Gonzalez-Fierro A., Dueñas-González A. (2018). Orlistat as a fasn inhibitor and multitargeted agent for cancer therapy. Expert Opin. Investig. Drugs.

[221] Kelly W., Diaz Duque A.E., Michalek J., Konkel B., Caflisch L., Chen Y., Pathuri S.C., Madhusudanannair-Kunnuparampil V., Floyd J., Brenner A. (2023). Phase ii investigation of tvb-2640 (denifanstat) with bevacizumab in patients with first relapse high-grade astrocytoma. Clin. Cancer Res..

[222] Datta S., Sears T., Cortopassi G., Woolard K., Angelastro J.M. (2021). Repurposing fda approved drugs inhibiting mitochondrial function for targeting glioma-stem like cells. Biomed. Pharmacother..

[223] Cui X., Jin Y., Hofseth A.B., Pena E., Habiger J., Chumanevich A., Poudyal D., Nagarkatti M., Nagarkatti P.S., Singh U.P. (2010). Resveratrol suppresses colitis and colon cancer associated with colitis. Cancer Prev. Res..

[224] Yue W., Hamaï A., Tonelli G., Bauvy C., Nicolas V., Tharinger H., Codogno P., Mehrpour M. (2013). Inhibition of the autophagic flux by salinomycin in breast cancer stem-like/progenitor cells interferes with their maintenance. Autophagy.

[225] Babaei G., Aziz S.G.-G., Jaghi N.Z.Z. (2021). Emt, cancer stem cells and autophagy; the three main axes of metastasis. Biomed. Pharmacother..

[226] Nath A., Li I., Roberts L.R., Chan C. (2015). Elevated free fatty acid uptake via cd36 promotes epithelial-mesenchymal transition in hepatocellular carcinoma. Sci. Rep..

[227] Wang C., Xu C., Sun M., Luo D., Liao D.-f., Cao D. (2009). Acetyl-coa carboxylase-α inhibitor tofa induces human cancer cell apoptosis. Biochem. Biophys. Res. Commun..

[228] To K.K., Poon D.C., Wei Y., Wang F., Lin G., Fu L.-w. (2015). Vatalanib sensitizes abcb1 and abcg2-overexpressing multidrug resistant colon cancer cells to chemotherapy under hypoxia. Biochem. Pharmacol..

[229] Orlando U.D., Garona J., Ripoll G.V., Maloberti P.M., Solano A.R., Avagnina A., Gomez D.E., Alonso D.F., Podesta E.J. (2012). The functional interaction between acyl-coa synthetase 4, 5-lipooxygenase and cyclooxygenase-2 controls tumor growth: A novel therapeutic target. PLoS ONE.

[230] Zhong L., Li Y., Xiong L., Wang W., Wu M., Yuan T., Yang W., Tian C., Miao Z., Wang T. (2021). Small molecules in targeted cancer therapy: Advances, challenges, and future perspectives. Signal Transduct. Target. Ther..

[231] Wu X., Dong Z., Wang C.J., Barlow L.J., Fako V., Serrano M.A., Zou Y., Liu J.-Y., Zhang J.-T. (2016). Fasn regulates cellular response to genotoxic treatments by increasing parp-1 expression and DNA repair activity via nf-κb and sp1. Proc. Natl. Acad. Sci. USA.

[232] Cotte A.K., Aires V., Fredon M., Limagne E., Derangère V., Thibaudin M., Humblin E., Scagliarini A., de Barros J.-P.P., Hillon P. (2018). Lysophosphatidylcholine acyltransferase 2-mediated lipid droplet production supports colorectal cancer chemoresistance. Nat. Commun..

[233] Xu J., Zhao W., Sun J., Huang Y., Wang P., Venkataramanan R., Yang D., Ma X., Rana A., Li S. (2018). Novel glucosylceramide synthase inhibitor based prodrug copolymer micelles for delivery of anticancer agents. J. Control. Release.

[234] Ohl K., Tenbrock K. (2018). Reactive oxygen species as regulators of mdsc-mediated immune suppression. Front. Immunol..

[235] Adeshakin A.O., Liu W., Adeshakin F.O., Afolabi L.O., Zhang M., Zhang G., Wang L., Li Z., Lin L., Cao Q. (2021). Regulation of ros in myeloid-derived suppressor cells through targeting fatty acid transport protein 2 enhanced anti-pd-l1 tumor immunotherapy. Cell. Immunol..

[236] Zhao F., Xiao C., Evans K.S., Theivanthiran T., DeVito N., Holtzhausen A., Liu J., Liu X., Boczkowski D., Nair S. (2018). Paracrine wnt5a-β-catenin signaling triggers a metabolic program that drives dendritic cell tolerization. Immunity.

[237] Shang L., Chen S., Du F., Li S., Zhao L., Wang X. (2011). Nutrient starvation elicits an acute autophagic response mediated by ulk1 dephosphorylation and its subsequent dissociation from ampk. Proc. Natl. Acad. Sci. USA.

[238] Liu L., Yan L., Liao N., Wu W.-Q., Shi J.-L. (2020). A review of ulk1-mediated autophagy in drug resistance of cancer. Cancers.

[239] Tang F., Hu P., Yang Z., Xue C., Gong J., Sun S., Shi L., Zhang S., Li Z., Yang C. (2017). Sbi0206965, a novel inhibitor of ulk1, suppresses non-small cell lung cancer cell growth by modulating both autophagy and apoptosis pathways. Oncol. Rep..

[240] Suvorova I.I., Pospelov V.A. (2019). Ampk/ulk1-dependent autophagy as a key mtor regulator in the context of cell pluripotency. Cell Death Dis..

[241] Brun S., Bestion E., Raymond E., Bassissi F., Jilkova Z.M., Mezouar S., Rachid M., Novello M., Tracz J., Hamaï A. (2022). Gns561, a clinical-stage ppt1 inhibitor, is efficient against hepatocellular carcinoma via modulation of lysosomal functions. Autophagy.

[242] Geisslinger F., Müller M., Vollmar A.M., Bartel K. (2020). Targeting lysosomes in cancer as promising strategy to overcome chemoresistance—A mini review. Front. Oncol..

[243] Mandhair H.K., Arambasic M., Novak U., Radpour R. (2020). Molecular modulation of autophagy: New venture to target resistant cancer stem cells. World J. Stem Cells.

[244] Peitzsch C., Gorodetska I., Klusa D., Shi Q., Alves T.C., Pantel K., Dubrovska A. (2022). Metabolic Regulation of Prostate Cancer Heterogeneity and Plasticity.

